# Reconstruction of the High-Osmolarity Glycerol (HOG) Signaling Pathway from the Halophilic Fungus *Wallemia ichthyophaga* in *Saccharomyces cerevisiae*

**DOI:** 10.3389/fmicb.2016.00901

**Published:** 2016-06-13

**Authors:** Tilen Konte, Ulrich Terpitz, Ana Plemenitaš

**Affiliations:** ^1^Faculty of Medicine, Institute of Biochemistry, University of LjubljanaLjubljana, Slovenia; ^2^Department of Biotechnology and Biophysics, Biocenter, Julius Maximilian University of WürzburgWürzburg, Germany

**Keywords:** signaling, protein-protein interaction, protein phosphorylation, mitogen activated protein kinase (MAPK), high-osmolarity glycerol (HOG) signaling pathway, *Saccharomyces cerevisiae*, halophilic fungus, *Wallemia ichthyophaga*

## Abstract

The basidiomycetous fungus *Wallemia ichthyophaga* grows between 1.7 and 5.1 M NaCl and is the most halophilic eukaryote described to date. Like other fungi, *W. ichthyophaga* detects changes in environmental salinity mainly by the evolutionarily conserved high-osmolarity glycerol (HOG) signaling pathway. In *Saccharomyces cerevisiae*, the HOG pathway has been extensively studied in connection to osmotic regulation, with a valuable knock-out strain collection established. In the present study, we reconstructed the architecture of the HOG pathway of *W. ichthyophaga* in suitable *S. cerevisiae* knock-out strains, through heterologous expression of the *W. ichthyophaga* HOG pathway proteins. Compared to *S. cerevisiae*, where the Pbs2 (ScPbs2) kinase of the HOG pathway is activated via the SHO1 and SLN1 branches, the interactions between the *W. ichthyophaga* Pbs2 (WiPbs2) kinase and the *W. ichthyophaga* SHO1 branch orthologs are not conserved: as well as evidence of poor interactions between the WiSho1 Src-homology 3 (SH3) domain and the WiPbs2 proline-rich motif, the absence of a considerable part of the osmosensing apparatus in the genome of *W. ichthyophaga* suggests that the SHO1 branch components are not involved in HOG signaling in this halophilic fungus. In contrast, the conserved activation of WiPbs2 by the *S. cerevisiae* ScSsk2/ScSsk22 kinase and the sensitivity of *W. ichthyophaga* cells to fludioxonil, emphasize the significance of two-component (SLN1-like) signaling via Group III histidine kinase. Combined with protein modeling data, our study reveals conserved and non-conserved protein interactions in the HOG signaling pathway of *W. ichthyophaga* and therefore significantly improves the knowledge of hyperosmotic signal processing in this halophilic fungus.

## Introduction

The hypersaline environments of salterns are inhabited by fungal species that are well adapted to these extreme environmental conditions (Gostinčar et al., 2011). Studies on these halophilic and halotolerant fungi aim to better understand the molecular mechanisms behind their extreme osmotolerance (Plemenitaš et al., 2014), which might then be applicable to the production of salt and drought resistant crops (Cominelli and Tonelli, 2010; Gašparič et al., 2013). The dominant fungal group in salterns is the polymorphic ascomycetous black yeasts (Zajc et al., 2012). However, the basidiomycete *Wallemia ichthyophaga*, which was originally isolated from the solar salterns of Sečovlje, Slovenia, is the most halophilic eukaryote (Zalar et al., 2005). This obligate halophilic fungus forms multicellular clumps in liquid media, has a coccoid, sarcina-like, morphology (Kralj Kunčič et al., 2010), and grows in media enriched with NaCl concentrations from 1.7 to 5.1 M. This phenotype is unusual in the fungal kingdom, and makes *W. ichthyophaga* an interesting model organism.

Recently, the osmoadaptation strategy of *W. ichthyophaga* has been investigated in detail. The NaCl range from 2.5 to 3.4 M was shown to be optimal for *W. ichthyophaga*, and associated with its highest dry biomass production (5–6 mg per ml of growth medium, respectively; Zajc et al., 2014). Despite these high external NaCl concentrations, *W. ichthyophaga* maintains low intracellular sodium concentrations, which only transiently increase upon hypersaline shock (Zajc et al., 2014). In *W. ichthyophaga*, glycerol is synthesized as a compatible solute for turgor regulation, and its concentration rises with increasing external salinity, and lowers under hypo-osmotic shock (Zajc et al., 2014). Also, at 4.3 M external NaCl concentrations, thickening of the cell wall, increased clump size, and higher abundance of extracellular polysaccharides appear to be important adaptations to the extreme external salinity for this fungus (Kralj Kunčič et al., 2010, 2013). The genome of *W. ichthyophaga* is at 9.6 Mb highly compact and contains only 4884 predicted protein-coding genes. Of these genes, 425 were differentially expressed when *W. ichthyophaga* was grown at 1.7 M, and 214 when it was grown at 5.1 M NaCl concentrations. The analysis of the transcriptomic data also revealed enrichment and salt-responsiveness of the hydrophobins, which are small cell-wall proteins usually involved in rigidity, strength and permeability, and have been hypothesized to be important in highly saline environments (Zajc et al., 2013).

For successful osmoadaptation in highly saline environments, it is essential that organisms can sense and respond to increase or decrease in salt concentrations (Plemenitaš et al., 2014). In yeast and fungi, increases in environmental osmolarity are sensed by the evolutionarily conserved high-osmolarity glycerol (HOG) signaling pathway (Krantz et al., 2006b; Bahn, 2008), which regulates the response of cells to new osmotic conditions (Hohmann et al., 2007). The HOG pathway has been extensively studied in *Saccharomyces cerevisiae*, where the final MAPK ScHog1 is phosphorylated upon hyperosmotic shock, and regulates several intracellular systems (Westfall et al., 2008; Mas et al., 2009; Sole et al., 2011), including, most importantly, glycerol production (Petelenz-Kurdziel et al., 2011). Once the cellular water potential is restored, the phosphatases ScPtc1, ScPtp2, and ScPtp3 can deactivate ScHog1 by its dephosphorylation (Hohmann et al., 2007). In *S. cerevisiae*, this pathway consists of two branches that are known as the SHO1 branch and the SLN1 branch. For the SHO1 branch, the signal is perceived by the combined actions of the transmembrane co-osmosensor ScSho1, the membrane anchor ScOpy2, and the mucines ScMsb2 and ScHkr1. Moreover, the guanine exchange factor ScCdc24 and the GTPase ScCdc42 are also involved in the SHO1 sensory apparatus. Signal perception leads to the activation of ScSte20 and ScCla4, which consequently activate the MAPKKK ScSte11. Redundantly, the SLN1 branch transduces the signal via the evolutionarily conserved two-component phosphorelay system, which consists of the ScSln1 sensor histidine kinase, the ScYpd1 intermediate protein, and the ScSsk1 response regulator that activates the MAPKKKs ScSsk2/ScSsk22 (Saito and Posas, 2012). The MAPKKKs ScSte11 and ScSsk2/ScSsk22 all activate the scaffold MAPKK ScPbs2. As ScPbs2 is the connecting hub of the two upstream pathway branches (i.e., SHO1, SLN1) and is an activator of ScHog1 MAPK, various interaction motifs, binding regions, localization signals, and the catalytic function are encoded in its sequence (Krantz et al., 2006a).

In filamentous fungi like *Aspergillus fumigatus* and *Aspergillus nidulans*, the HOG pathway is also involved in osmotic stress responses (Ma and Li, 2013; de Oliveira Bruder Nascimento et al., 2016). However, in *A. nidulans*, the ShoA (AnSho1) protein is not involved in osmosensing (Furukawa et al., 2005). In a similar manner, the HOG pathway in the basidiomycetous fungus *Cryptococcus neoformans* is activated exclusively via the two-component phosphorelay system (Bahn et al., 2006). In addition to osmotic regulation, the two-component branches of the HOG pathway in pathogenic *C. neoformans* (Kozubowski et al., 2009), *Magnaporthe oryzae* (Jacob et al., 2015), and *Candida albicans* (Prieto et al., 2014) are associated with virulence regulation, plant infection, and colonization, respectively. In these pathogens, the pathway is regulated mainly by histidine kinases, which also confer fungicide sensitivity (Kozubowski et al., 2009; El-Mowafy et al., 2013).

The HOG pathway was recently described in detail in halotolerant *Hortaea werneckii*, where survival of the cells depends on the HOG pathway only at salinities ≥3.0 M NaCl (Kejžar et al., 2015). Initial studies of the HOG pathway in *W. ichthyophaga* showed that its regulation is controlled by rapid dephosphorylation under both hyperosmolar and hypo-osmolar stress (Konte and Plemenitaš, 2013). Interestingly, the *W. ichthyophaga* genome contains two HOG1-like genes: *WiHOG1A* and *WiHOG1B*, which are up-regulated three- and six-fold, respectively, under both hyper- and hypo-osmolar conditions. Results from our previous complementation studies with the *WiHOG1A* and *WiHOG1B* genes in *S. cerevisiae* showed that while the WiHog1B protein was able to fully rescue the *hog1*Δ cells, WiHog1A showed a partial rescue of the osmosensitive phenotype (Konte and Plemenitaš, 2013).

In the current study, we further probed the HOG pathway of *W. ichthyophaga* using *S. cerevisiae* HOG pathway mutants, similar to our previous investigations (Konte and Plemenitaš, 2013). The *S. cerevisiae* mutants are easily grown, genetically tractable, and a large library of knock-out mutant strains is available. In particular, the high conservation of the MAPK signaling pathway components makes *S. cerevisiae* an ideal system for functional investigations of orthologous signaling proteins from fungi (Bansal et al., 2001; El-Mowafy et al., 2013), and even from evolutionarily distant organisms such as plants (Reiser et al., 2003; Jain et al., 2009) and mammals (Levin-Salomon et al., 2009). Here, we have employed the above system to further elucidate the HOG pathway components in halophilic *W. ichthyophaga*.

## Materials and methods

### Strains, growth conditions, DNA and RNA isolation, and cDNA synthesis

*W. ichthyophaga* (EXF 994) was provided by the culture collection of the Department of Biology, Biotechnical Faculty, University of Ljubljana (EXF), and was grown at 28°C and 180 rpm in YNB medium supplemented with 3.4 M NaCl, as described previously (Konte and Plemenitaš, 2013). The *S. cerevisiae* parental strains (here referred to as the wild-types; WT) and mutant strains used in this study are listed in Supplemental Table S1. For the detailed growth conditions of these *S. cerevisiae* strains, please see the section on “*Expression of Genes in the S. cerevisiae Mutants and Halotolerance Assays*” below. A method described previously (Rozman and Komel, 1994) was used for isolation of genomic DNA from *W. ichthyophaga* cells in mid-exponential-phase grown in YNB medium with 3.4 M NaCl, and from the Euroscarf *S. cerevisiae* Y00000 (S288c) WT strain grown in YNB medium. The construction of pools of uncloned, adaptor-ligated, genomic DNA fragments of *W. ichthyophaga* was carried out with Genome Walker Universal kits (Clontech). The mid-exponential-phase cells of *W. ichthyophaga* grown in YNB medium containing 3.4 M NaCl were also used for the RNA extraction, with the TRI Reagent (Sigma Aldrich). The DNA was removed using DNAse I (Fermentas), and the RNA was evaluated spectrophotometrically (A260/A280, BioTek Synergy 2 Take3 plates) and by capillary electrophoresis (Agilent 2100 bioanalyser). SuperScript III First-Strand cDNA Synthesis kits (Invitrogen) and random hexamer primers (Promega) or SMARTer Race cDNA Amplification kits (Clontech) were used for the synthesis of cDNA.

### Identification, amplification, cloning, and sequencing of the genes under study

A partial sequence of the *PBS2*-like gene from *W. ichthyophaga* was cloned in our previous study (Lenassi et al., 2007) using degenerate primers. In the present study, the partial sequence of the *WiSTE11* gene was obtained using the same method. The degenerate primers degWiSTE11.f and degWiSTE11.r were constructed based on the highly conserved parts of the WiSte11 kinase domain (GELMAVKQV, NIFLEYVPG) using the iCODEHOP software, and then manually refined. Gene-specific primers were designed (see Supplemental Table S2: the pWiPBS2 and npWiPBS2, and pWiSTE11 and npWiSTE11 primers) based on the partial sequences of the *WiSTE11* and *WiPBS2* genes. These primers were used in the primary touch-down PCR and the following secondary nested PCR (annealing for 5 cycles at 70°C, 5 cycles at 68°C, and 25 cycles at 68°C), which was performed with High Fidelity Polymerase Mix (Fermentas) and 50 ng cDNA or gDNA template, in 20-μL reactions. The amplicons were purified with EZNA gel extraction kits (Omega Bio-Tek), cloned into the pJET cloning vector (Fermentas), transformed into *E. coli* XL-1 Blue cells, isolated with GeneJET MiniPrep kits (Fermentas), and sequenced (Macrogen). The sequences were later confirmed in the genome of *W. ichthyophaga* that became available during the present study (Zajc et al., 2013). The *ScPBS2* and *ScSTE11* gene sequences were retrieved from the *Saccharomyces* Genome Database (SGD Project; http://www.yeastgenome.org/; March 2015) and were cloned using the same method. The *WiSHO1* gene sequence was obtained using local BLAST searches of the *W. ichthyophaga* genome (Zajc et al., 2013). The pAZ301 vector (pR316 with *SHO1* promoter and *GFP* tag) containing the *ScSHO1* gene was kindly provided by W. Lim (UCSF, San Francisco). The *WiHOG1A* (GenBank accession number AGG39582), *WiHOG1B* (GenBank accession number AGG39583) and *ScHOG1* (GenBank accession number DAA09427) genes were amplified and cloned in our previous study (Konte and Plemenitaš, 2013). Based on the sequences obtained, new forward and reverse primers were designed for PCR amplification of the whole gene-coding sequences, again with 50 ng template cDNA, High Fidelity Polymerase Mix, and annealing for 30 cycles at 60°C. Again, the amplicons were purified, cloned, and sequenced.

### Sequences, domains, and phylogenetic analysis

For protein sequence alignments, the M-Coffee software with default settings was used (http://tcoffee.crg.cat/apps/tcoffee/do:mcoffee; July 2015) and visualization was performed with the Jalview 2.7 software (Waterhouse et al., 2009). Annotation of domains in alignment was based on the literature and data from the UniProt database (Uniprot Consortium, 2012; http://www.uniprot.org/; July 2015) and the SMART software (http://smart.embl-heidelberg.de/; June 2015). The proline-rich motifs of WiPbs2 and ScPbs2 were annotated according to the Eukaryotic Linear Motif Resource (http://elm.eu.org/; July 2015) and the literature. The physicochemical parameters of the protein sequences were obtained with the ProtParam ExPasy program (http://web.expasy.org/protparam/; June 2015; Wilkins et al., 1999). Sequences of the orthologous Pbs2 kinases were retrieved from the National Center for Biotechnology Information (NCBI) database; (http://www.ncbi.nlm.nih.gov/; April 2015). For identification of the *W. ichthyophaga* HOG pathway components, the *W. ichthyophaga* genome was searched for orthologous pathway proteins with the local BLAST software (version 2.2.25; http://blast.ncbi.nlm.nih.gov/Blast.cgi?CMD=Web&PAGE_TYPE=BlastDocs&DOC_TYPE=Download; November 2010) using as probes the *S. cerevisiae* HOG pathway proteins, and the *C. neoformans* histidine kinase Nik1 (see Supplemental Table S3 for GenBank accession numbers). The NCBI Conserved Domain Database (CDD; Marchler-Bauer and Bryant, 2004) was also the source of domain annotations of the identified *W. ichthyophaga* HOG pathway. The maximum likelihood phylogenetic tree of orthologous proteins was calculated as described before (Konte and Plemenitaš, 2013). The interactions of the Src homology 3 (SH3) domain from WiSho1 and the WiPbs2 proline-rich motif were modeled with the SWISS-MODEL software (Biasini et al., 2014), based on the crystal structure of the ScSho1 SH3 domain complexed with the ScPbs2 proline-rich motif (PDB: 2VKN). In the model, the ScPbs2 proline-rich motif was replaced with the WiPbs2 APLPPNQLR peptide using the WhatIf program (Vriend, 1990). The COCOMAPS software (Vangone et al., 2011) was used to analyze the residue interactions in the models and the PyMOL software was used for visualization of the three-dimensional structures (The PyMOL Molecular Graphics System, Version 1.1, LLC).

### Expression of genes in the *S. cerevisiae* mutants, and halotolerance assays

The expression of the *W. ichthyophaga WiPBS2* and *WiSTE11* genes in *S. cerevisiae* cells was carried out with the corresponding open reading frame amplification from the pJET cloning vectors (see Section “Identification, Amplification, Cloning, and Sequencing of the Genes under Study,” above) with primers containing restriction sites (Supplemental Table S2). The resulting products were cloned into the corresponding restriction sites of the low-copy-number plasmids pYX142 (*CEN, LEU, TPI* promoter, *Amp*^r^), YCplac22 (*CEN, TRP, TPI* promoter, *Amp*^r^), and YCplac 33 (*CEN, URA, TPI* promoter, *Amp*^r^), and transformed into the *S. cerevisiae* strains using the Quick and Easy LiAc/SS carrier DNA/ polyethylene glycol method (Gietz and Schiestl, 2007). The open reading frame of the *WiSHO1* gene was amplified from the pJET cloning vector, with the primers WiSHO1xSHO.r and GFPxWiSHO1.r, which have sequence overhangs that are homologous to the 3′ end of the *SHO1* promoter and the 5′ end of the GFP tag. The *SHO1* promoter of *S. cerevisiae* and the GFP tag were amplified separately from the pAZ301 plasmid using the primers (KpnI)SHO.f and WiSHO1xSHO.r, and WiSHO1xGFP.f and (SacI)GFP.r, respectively. The amplified *SHO1* promoter, the *WiSHO1* gene, and the GFP tag were then combined in the PCR reaction with High Fidelity Polymerase Mix and the primers (KpnI)SHO.f and (SacI)GFP.r, and annealed for 30 cycles at 60°C. The product was confirmed by sequencing, and cloned into pAZ301 (*CEN, URA, Amp*^r^), which was previously cut with *Kpn*I and *Sac*I to remove the *ScSHO1*. In addition to *WiSHO1*, we also constructed and cloned the hybrid gene between *ScSHO1* and *WiSHO1* into pAZ301. This hybrid gene was made from *ScSHO1*, where the part of the sequence that encodes the *ScSHO1* SH3 domain was replaced with the sequence of the *WiSHO1* SH3 domain [amplified with primers (BamHI)WiSH3.f and (EcoRI)WiSH3.r; Supplemental Table S2], using the *BamH*I and *EcoR*I restriction sites already present in *ScSHO1*. The constructed hybrid gene is named as *ScSHO1WiSH3*. The resulting plasmid constructs and transformants are given in Supplemental Table S4. For controls, both the mutant strains expressing *S. cerevisiae* genes (*ScPBS2, ScSHO1, ScSTE11, ScHOG1*) and WT strains or mutant strains transformed with empty vectors (EVs) were used. The cells were grown on YNB plates with appropriate drop-out selection (Supplemental Table S4), with the presence of the plasmids confirmed by colony PCR.

For the construction of the WiPbs2 protein with the inserted proline-rich motif VNKPLPPLPVAG from ScPbs2, we used the oligonucleotide-directed PCR mutagenesis method. Using the primers WiPbs2(ScPRO).f and r (Supplemental Table S2) and the plasmid YCplac22+*WiPBS2*, the ScPbs2 proline-rich motif was inserted using a PCR reaction with annealing for 30 cycles at 60°C. The resulting plasmid YCplac22+*WiPBS2ScPRO* was transformed simultaneously with the plasmid pAZ301+*ScSHO1WiSH3* into the *sho1*Δ*ssk2/22*Δ*pbs2*Δ cells. Furthermore, the simultaneous transformation of YCplac22+*WiPBS2* and pAZ301+*ScSHO1WiSH3*, YCplac22+*ScPBS2*, and pAZ301+*ScSHO1*, or YCplac22+*ScPBS2* and EV pAZ301 into the *sho1*Δ*ssk2/22*Δ*pbs2*Δ cells was also performed. Positive transformants were confirmed by PCR and then used for the halotolerance assays. They were grown to mid-exponential phase in YNB medium containing: 0.08% (w/v) Complete Supplement Mixture with appropriate drop-out (Formedium), 0.16% (w/v) YNB (Formedium), 2.0% (w/v) glucose (Mallinckrodt Baker), 0.5% (w/v) ammonium sulfate (Carlo Erba Reagents), and NaCl (Carlo Erba Reagents) added to different concentrations, at pH 7.0 and 30°C in a rotary shaker (180 rpm). The OD_600_ was then adjusted to 0.5, and 3.5 μL of 10-fold serial dilutions (1–10^3^ dilutions) were spotted onto the selective plates (YNB with the appropriate drop-out, and 2% (w/v) agar) that contained the indicated NaCl concentrations. We incubated the plates at 30°C for 3–6 days, and then scanned them. All of the experiments were repeated 2–3 times.

### The cross-talk β-galactosidase assay

The activation of Fus3 kinase was followed under salt stress with the induction of the cross-talk mating response of the *FUS1-lacZ* reporter expression (O'Rourke and Herskowitz, 1998). The *FUS1-lacZ* reporter system was integrated into the genome of *S. cerevisiae pbs2*Δ*hog1*Δ cells using yeast integration plasmids pRS306N (*URA3* integration) and into the *ssk2/22*Δ*pbs2*Δ cells using pRS303H (*HIS3* integration). The *FUS1-lacZ* reporter system integration with pRS306N was also performed for the wild-type S1278b cells, which were used as the negative control for *pbs2*Δ*hog1*Δ *FUS1-lacZ*. For the negative control of the *ssk2/22*Δ*pbs2*Δ *FUS1-lacZ* cells, the W303 WT strain was transformed with the YCplac22 vector containing the *FUS1-lacZ* reporter. Next, in *pbs2*Δ*hog1*Δ *FUS1-lacZ* cells, WiPbs2 or ScPbs2 and WiHog1A, WiHog1B, or ScHog1 were expressed, and in *ssk2/22*Δ*pbs2*Δ *FUS1-lacZ* cells WiPbs2 or ScPbs2 were expressed. For both strains, the EV (positive control) was also transformed (see Supplemental Table S4 for detailed description of these transformants). The positive transformants were subjected to 1 M NaCl for 4 h, and the activity of the β-galactosidase enzyme before and after the application of NaCl was measured, as described previously (Konte and Plemenitaš, 2013). We calculated the β-galactosidase values according to the formula (A_420_× 1000 − 1.75 × OD_550_)/(time_(*min*)_× V_(*mL*)_× OD_600_) (Miller, 1972; Murakami et al., 2008), in triplicate, and the means ± SD were calculated. The graphs were designed using the GraphPad Prism 5 software.

#### Western blotting

For the Western blotting, mid-exponential-phase *pbs2*Δ*hog1*Δ cells expressing WiPbs2 or ScPbs2 and one of the kinases WiHog1A, WiHog1B, or ScHog1 were adjusted to an OD_600_ of 0.5. The transformants were harvested by centrifugation (6000 × *g*, 1 min, 25°C) before and 5 min and 10 min after application of 0.7 M NaCl, and frozen in liquid nitrogen. Protein extracts were prepared with the boiling of cells in protein extraction buffer (100 mM Tris-HCl, pH 6.8, 20% glycerol, 2% 2-mercaptoethanol, 4% SDS) containing a cocktail of fungal protease inhibitors (Sigma Aldrich) and phosphatase inhibitors (Thermo Scientific), for 10 min. The BCA protein assay (Pierce) was used to measure protein concentrations, and equal amounts of proteins were boiled for 10 min in 5 × protein-loading buffer before loading (0.313 M Tris-HCl, pH 6.8 at 25°C, 10% SDS, 0.05% bromophenol blue, 50% glycerol; Fermentas). After separation by SDS–PAGE in 12% polyacrylamide gels, the protein extracts were blotted onto Immobilon PVDF membranes (Millipore) and incubated with: anti-phospho-p38 antibodies (Rabbit, Cell Signaling, LOT21, #9211BC) for phosphorylated Hog1; anti-MYC-tag antibodies (Rabbit, Abcam, ab9106) for tagged Pbs2; and anti-rabbit secondary antibodies (Cell Signaling Technology) conjugated with horseradish peroxidase. The Rapid Step ECL Reagent (Calbiochem) and a LAS-1000 camera (Fuji) were used for visualization of the signals on the membranes.

### Fluorescence microscopy

The EVs and plasmids pAZ301+*ScSHO1*, pAZ301+*WiSHO1*, and pAZ301+*ScSHO1WiSH3* where the proteins were C-terminally tagged with GFP, were transformed into *S. cerevisiae sho1*Δ*ssk2/22*Δ cells. Positive transformants were grown in YNB-URA medium to mid-exponential growth phase in a rotary shaker (180 rpm, 30°C), and the OD_600_ was adjusted to 0.5. Before the preparation of microscope slides, the cells were exposed to 0.5 M NaCl for 5 min, washed three times in PBS, fixed in 3% paraformaldehyde, and resuspended in distilled water. Then the cells were dried onto poly-D-lysine–coated slides, mounted with Vectashield (Vector Laboratories), and imaged at room temperature with a camera (AxioCam Mrm) on an Axio Imager M2 (Carl Zeiss), equipped with a Plan-Apochromat objective (63 × /1.4), and an illuminator (HXP 120 C). For each field, GFP images (excitation, 488 nm; detection, 505–530 nm) and differential interference contrast images were captured using the ZEN 2012 software (Carl Zeiss).

### Fludioxonil sensitivity assays

The cells of *W. ichthyophaga* were grown to mid-exponential phase at 28°C and 180 rpm in YNB medium supplemented with 3.4 M NaCl (Carlo Erba Reagents). Then 30 mL cell culture was harvested by centrifugation (4600 × *g*, 10 min, 25°C), and the pellet was washed twice with 30 mL modified OS buffer (50 mM succinic acid, 1 M NaCl, 2.75 M sorbitol, pH 7.5) using the same centrifugation parameters. The pellet was then resuspended in 10 mL modified OS buffer that contained 32 mg cell-wall-degrading enzyme cocktail Caylase (Cayla), and incubated at 25°C and 50 rpm for 1.5 h to disintegrate cellular clumps. Next, the cells were harvested and resuspended in 5 mL YNB containing 2.75 M sorbitol, and then they were 10-fold diluted in YNB containing 2.75 M sorbitol, 100 μg/mL fludioxonil (Sigma Aldrich), and 0.75% low-melting point agarose, and poured onto YNB agar plates that contained different NaCl concentrations (i.e., 2.5, 3.4, 4.3 M) and 100 μg/mL fludioxonil. The plates were incubated at 28°C and their growth was scanned after 7–10 days.

## Results

### Identification of orthologous hog-pathway proteins in the genome of *W. ichthyophaga*

**Figure 1:**
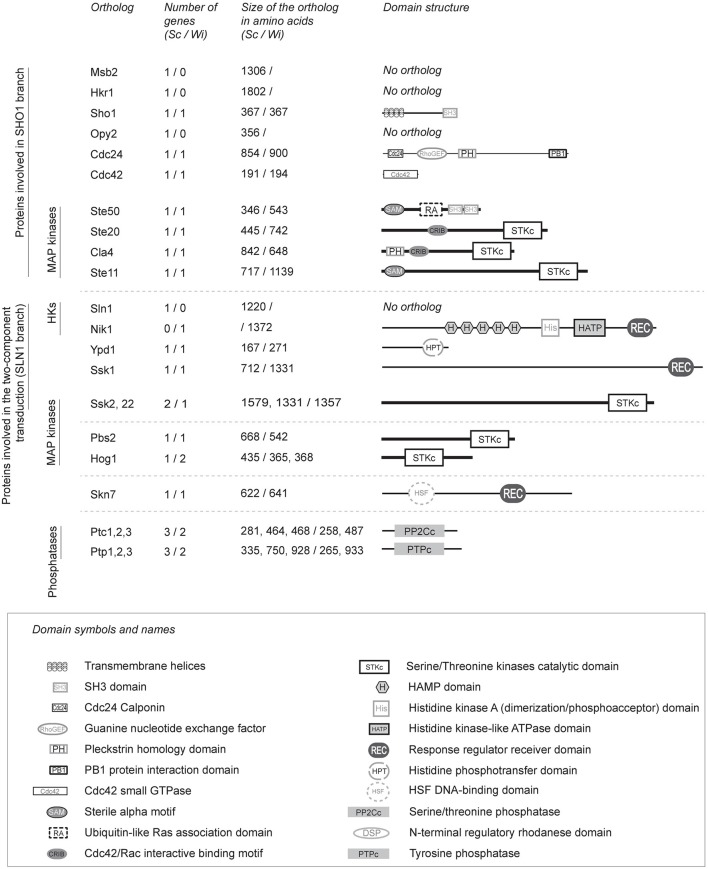
**Details of the orthologous proteins of the ***S. cerevisiae*** HOG signaling pathway in ***W. ichthyophaga*****. The HOG signaling proteins were identified in the genome of *W. ichthyophaga* with the local BLAST algorithm using known HOG pathway proteins as probes (Supplemental Table S3). Sc (*S. cerevisiae*) and Wi (*W. ichthyophaga*) in column headers indicate the source organism of the data. The lengths of the illustrated domain structures correspond to the actual sizes of the orthologs. HKs, histidine kinases. For the GenBank accession numbers of *W. ichthyophaga* proteins, please see Supplemental Table S5. Domain annotations were retrieved from NCBI CDD (Marchler-Bauer and Bryant, 2004), according to: SH3 domain, pfam00018; Cdc24 calponin, pfam06395; guanine nucleotide exchange factor, cd00160; pleckstrin homology domain, cd13246; PB1 protein interaction domain, cd05992; Cdc42 small GTPase, cd01874; Sterile alpha motif, cd09533; Ubiquitin-like Ras-association domain, cd01786; Cdc42/Rac-interactive binding motif, cd01093; serine/ threonine kinases catalytic domain, smart00220; HAMP domain, cd06225; histidine kinase A dimerization/phosphoacceptor domain, cd00082; histidine kinase-like ATPase domain, cd00075; response regulator receiver domain, pfam00072; histidine phosphotransfer domain, cd00088; HSF DNA-binding domain, pfam00447; serine/ threonine phosphatase, cd00143, tyrosine phosphatase, cd00047.

We searched for homologs of the *S. cerevisiae* HOG pathway in *W. ichthyophaga* (Figure 1). Additionally we also searched for a homolog of the histidine kinase Tco1 (CnNik1) as it has been shown to be involved in the HOG pathway of *C. neoformans* (Bahn et al., 2006). We were unable to identify homologs of the sensory mucins ScMsb2 and ScHkr1, and the anchor ScOpy2. However, we did identify homologs of the WiSho1, WiCdc24, WiCdc42, WiSte20, WiCla4, and WiSte11 proteins, which all show similar domain structures to the homologs from *S. cerevisiae*. Additionally, we determined that WiSte50 contains two SH3 domains, which are absent in ScSte50, in addition to the characteristic Ras-association domain (CDD accession number pfam00788) and Sterile alpha motif (CDD accession number cl15755; Figure 1; for GenBank accession numbers of *W. ichthyophaga* HOG-pathway proteins please see Supplemental Table S5).

The results from our BLAST searches showed that the core phosphorelay system of the SLN1-like branch is well conserved in *W. ichthyophaga*. This system contains WiYpd1 and WiSsk1, the kinase WiSsk2, and the response regulator WiSkn7 (its ortholog, ScSkn7, interacts with ScYpd1, and in *S. cerevisiae* it is involved in the hypo-osmotic response; Saito and Posas, 2012). However, we were unable to detect an ortholog of ScSln1 in *W. ichthyophaga*. Instead, we did find the Group III cytosolic histidine kinase, WiNik1, which contains the Histidine kinase, Adenylyl cyclase, Methyl-accepting protein, and Phosphatase (HAMP; CDD accession number cl01054) domain repeats. The genome of *S. cerevisiae* does not contain Nik1, but its orthologs are involved in osmosensing in other fungi (Bahn, 2008; El-Mowafy et al., 2013).

The HOG pathway in *S. cerevisiae* is regulated by negative-feedback through the action of phosphothreonine and phosphotyrosine phosphatases: ScPtc1, ScPtp2, and ScPtp3 (Hohmann et al., 2007). In the genome of *W. ichthyophaga*, we identified two phosphothreonine phosphatases—WiPtc1 (homolog of ScPtc1), and WiPtc3 (homolog of ScPtc3 and ScPtc2), as well as two phosphotyrosine phosphatases—WiPtp1 (homolog of ScPtp1), and WiPtp3 (homolog of ScPtp3, which is a broad-range phosphatase).

### Comparison of WiPbs2 with ScPbs2, and with other fungal Pbs2-like kinases

As Pbs2-like kinases are the central element of fungal HOG pathways (Krantz et al., 2006b), we further analyzed and compared WiPbs2 with ScPbs2 (the comparison is summarized in Table 1), to identify any conserved elements of this protein. The *WiPBS2* gene (GenBank accession number EOR04233) has 1725 nucleotides and contains two introns, one 47-bp-long (from nucleotide 1096–1142) and the other 49-bp-long (from nucleotide 1269–1317). We predict the remaining 1629 nucleotides to encode a 542 amino acid protein with a 542 amino acid protein with a theoretical isoelectric point (pI) of 6.96 and a predicted molecular weight of 59.26 kDa. Amino-acid alignment of the orthologous Pbs2-like kinases reveals poor conservation of the N-terminal region, but higher conservation of the C-terminal region. The C-terminus contains the kinase domain (Supplemental Figure S1, a region located within the two asterisks in the alignment), which starts six amino acids before the well-conserved ATP-binding site. High conservation was observed in the core of the kinase domain, especially in the active site and the activation loop that contains the SLAKT motif. This motif is important for the activation of the kinase, with its Ser and Thr residues phosphorylated by MAPKKK (Maeda et al., 1995; Supplemental Figure S1). Moreover, just after the end of the kinase domain in ScPbs2, there is a nuclear localization signal (Tatebayashi et al., 2003). This YITE sequence is poorly conserved in WiPbs2, as in other selected Pbs2 kinases (Supplemental Figure S1).

**Table 1:** **Comparison of the gene and protein attributes of WiPbs2 and ScPbs2**.

**Attribute**	**WiPbs2**	**ScPbs2**	**Reference for ScPbs2**
Gene length (base pairs)	1725	2007	*Saccharomyces* Genome Database
Number of introns	2	0	*Saccharomyces* Genome Database
CDS length (base pairs)	1629	2007	*Saccharomyces* Genome Database
Protein length (amino acids)	542^a^	668	*Saccharomyces* Genome Database
Isoelectric point	6.96^a^	9.44	*Saccharomyces* Genome Database
Molecular weight (kDa)	59.26^a^	72.73	*Saccharomyces* Genome Database
Kinase domain residues	244–501^a^	360–623	UniProtKB accession number P08018
ATP-binding site	250–258^a^	366–374	UniProtKB accession number P08018
Active site residue	Asp366^a^	Asp485	UniProtKB accession number P08018
Activation loop residues	383–404^a^	503–524	CDD accession number cd06622
Activation residues	Ser394, Thr398^a^	Ser514, Thr518	Maeda et al., 1995
Nuclear localization signal residues	Poorly conserved	636–639	Tatebayashi et al., 2003
Nuclear export signal residues	Ile8, Leu11^a^	Leu8, Leu10	Tatebayashi et al., 2003
Docking site for ScSsk2/ScSsk22	Poorly conserved	46–56	Tatebayashi et al., 2003
Proline-rich motif (Sho1 binding)	Poorly conserved	91–101	Maeda et al., 1995; Raitt et al., 2000; Marles et al., 2004
Docking site for ScSte11	Poorly conserved	55–107	Zarrinpar et al., 2004; Tatebayashi et al., 2006
ScHog1-interaction domain	Partially conserved	283–353	Zarrinpar et al., 2004; Murakami et al., 2008

a *Predicted or putative*.

**Figure 2:**
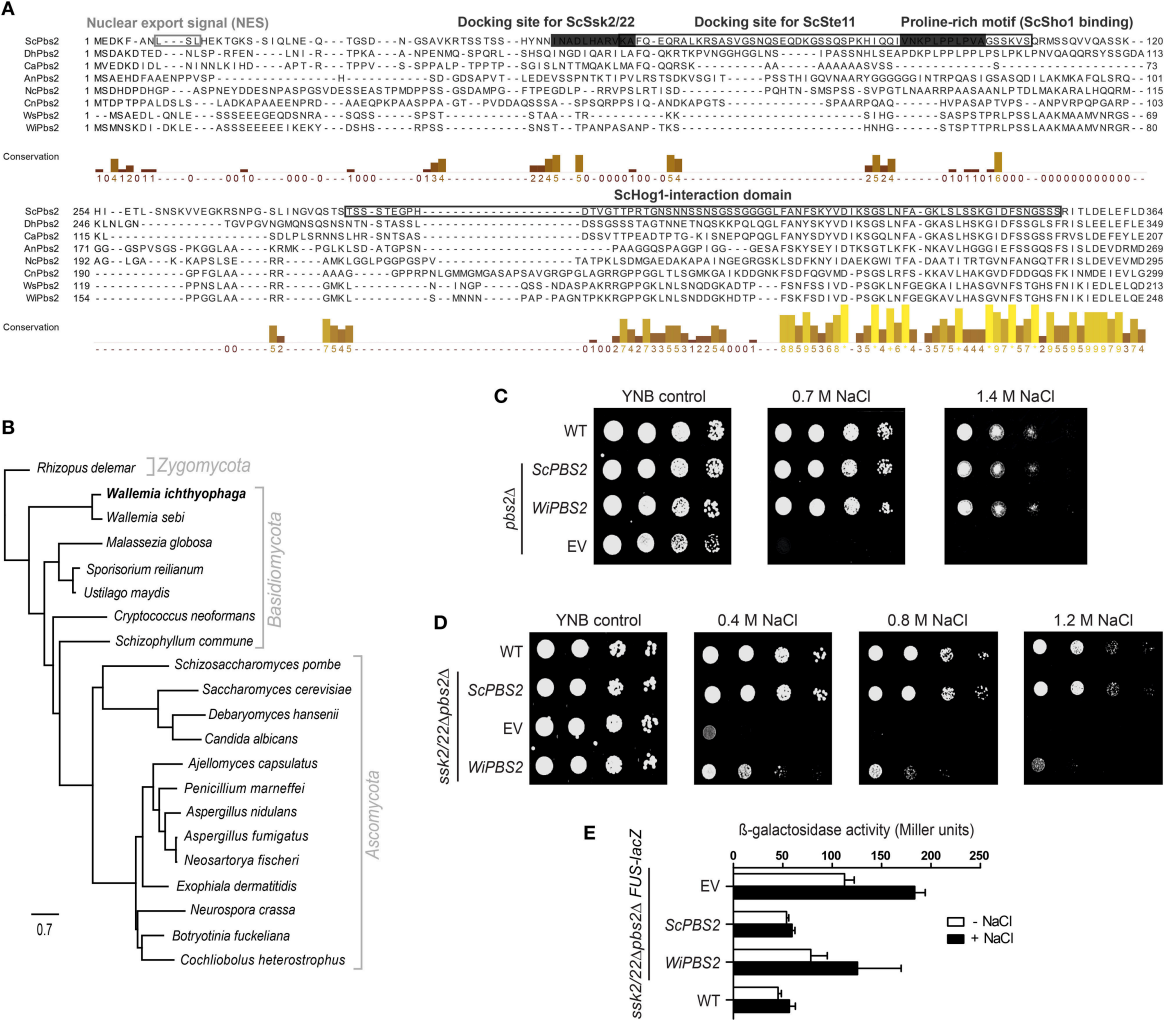
**Characterization of WiPbs2. (A)** Partial protein alignment of selected Pbs2 kinases. Prefixes indicate the source organism of Pbs2: Sc, *S. cerevisiae*; Dh, *D. hansenii*; Ca, *C. albicans*; An, *A. nidulans*; Nc, *N. crassa*; Cn, *C. neoformans*; Ws, *W. sebi*; Wi, *W. ichthyophaga*. Important domains, motifs and sites are highlighted. Framed boxes, ScPbs2 nuclear export signal, docking site for ScSte11, and ScHog1 interaction domain; gray boxes, ScPbs2 docking site for ScSsk2/ScSsk22, and proline-rich motifs for ScSho1 binding. The columns demonstrate conservation of amino acids (higher and lighter, greater conservation). See Supplemental Table S3 for the GenBank accession numbers. **(B)** Maximum likelihood phylogenetic tree of orthologous Pbs2 kinases arbitrarily rooted to *Rhizopus delemar*. **(C)** Representative halotolerance rescue assay. The *S. cerevisiae* Y00000 WT strain and *pbs2*Δ strain were transformed as the EV positive control (WT) and *pbs2*Δ negative control (EV), respectively, and *pbs2*Δ with *ScPBS2* (positive control) or *WiPBS2*. **(D)** Representative halotolerance rescue assay with the W303 wild-type strain (WT) and the *ssk2/22*Δ*pbs2*Δ strain transformed as in **(C). (E)** Cross-talk activation of the mating pathway, as β-galactosidase activity, in *ssk2/22*Δ*pbs2*Δ*FUS-lacZ* cells transformed as in **(C)**. Activities were measured before and after 1 M NaCl for 4 h. Data are means ± SD (*n* = 3). Box indicating docking site is to dark.

The N-terminal region of ScPbs2 contains important motifs for interactions with its protein partners (Figure 2A). ScPbs2 residues 4–18 contain a nuclear export signal, with indicative Leu residues at positions 8 and 10 (Tatebayashi et al., 2003). In WiPbs2, Ile at position 8 and Leu at position 11 might serve the same function. ScPbs2 residues 46–56 are important for SLN1 signaling and contain the ScSsk2/ScSsk22 docking site (Tatebayashi et al., 2003; Figure 2A). In agreement with earlier investigations, we observed that the Ala52, Arg53, and Ala56 motif is conserved in yeast; however, in many filamentous and basidiomycetous fungi it is not (Krantz et al., 2006b; Figure 2A). Moreover, the proline-rich motif of *S. cerevisiae* represented by residues 91–101, which is crucial for ScSho1 binding and signaling through the SHO1 branch (Maeda et al., 1995; Raitt et al., 2000; Marles et al., 2004; Figure 2A) is also well conserved in the majority of yeast (Krantz et al., 2006a). In contrast, we found that this motif is not conserved in either *W. ichthyophaga* or in the selected filamentous and basidiomycetous fungi shown in Figure 2A. Other important features in ScPbs2 are the docking site for ScSte11, which has been mapped to residues 55–107 (Zarrinpar et al., 2004; Tatebayashi et al., 2006) and overlaps with the ScSsk2/ScSsk22 docking site, and the ScHog1-interaction domain (Figure 2A). Our alignment revealed that the Ste11 docking site in Pbs2-like kinases from *W. ichthyophaga* and selected fungi is only poorly conserved (Figure 2A). Similarly, the region proximal to the kinase domain (residues 283–353), which in *S. cerevisiae* is required for signal transmission from ScPbs2 to ScHog1 (Zarrinpar et al., 2004; Murakami et al., 2008; Figure 2A, ScHog1-interaction domain), is only partially conserved in the aligned Pbs2 kinases. These sequences show notable conservation only downstream of the ScPbs2 Thr297 (Figure 2A).

These findings are supported by the maximum likelihood phylogenetic tree in which we compared the identified WiPbs2 sequence to orthologs of other fungi (Figure 2B). This phylogenetic tree was arbitrarily rooted to *Rhizopus delemar*. The yeasts grouped in a clade that is clearly separated from filamentous and other fungi. On the other hand, the MAPKK Pbs2 from *W. ichthyophaga* is the most similar to the MAPKK from *Wallemia sebi*, and as expected, these are both positioned at the base of Basidiomycota, near Zygomycota.

Although the ScPbs2-interaction motifs are not highly conserved in WiPbs2 kinase (Figure 2A) we wanted to examine if WiPbs2 can complement the function of the missing ScPbs2, when expressed in *S. cerevisiae pbs2*Δ cells (Figure 2C). The growth of the WiPbs2-expressing cells was similar to the cells expressing ScPbs2 and the WT cells, regardless of the salt concentration used (Figure 2C; 0.7 M NaCl, 1.4 M NaCl). The cells transformed with EV were used as the negative control, and as expected, their growth was only evident on the YNB-LEU plates (Figure 2C, YNB control). This showed that in the *pbs2*Δ background, WiPbs2 kinase can completely replace the function of the missing ScPbs2 when cells are grown in salt.

### WiPbs2 is not efficiently activated by the SHO1 branch in *S. cerevisiae*

In *S. cerevisiae*, the native ScPbs2 kinase can be activated by either the SHO1 or the SLN1 branch of the HOG pathway (Hohmann et al., 2007). Although the results from Figure 2C show that WiPbs2 fits into the HOG pathway of *S. cerevisiae* with no observed differences compared to ScPbs2, in these *pbs2*Δ cells it is not possible to discriminate through which branch the WiPbs2 kinase was activated (i.e., SHO1 or SLN1). Therefore, we next expressed the WiPbs2 kinase in *S. cerevisiae ssk2/22*Δ*pbs2*Δ cells. Due to the Ssk2/Ssk22 deletion in this strain, the SLN1 branch was inactive and the signal was transferred to the WiPbs2 kinase only via the SHO1 branch. We transformed the *ssk2/22*Δ*pbs2*Δ cells with WiPbs2 or ScPbs2, and plated them on YNB-TRP medium. The strain *ssk2/22*Δ*pbs2*Δ with EV was used as the negative control, and the WT strain as the positive control. The data presented in Figure 2D show that the growth of these cells expressing ScPbs2 was similar to the WT cells (Figure 2D, *ScPBS2*, WT) under all of the tested salinities. Compared to the ScPbs2-expressing and WT cells, the growth of the WiPbs2-expressing cells was reduced, and was almost completely inhibited at 1.2 M NaCl (Figure 2D, *WiPBS2*).

These data were confirmed with the β-galactosidase assay (Figure 2E). In these assays, we monitored the activation of Fus3 kinase in *S. cerevisiae* under salt stress, because the signal leaks from the HOG pathway to the mating pathway when some of the components are missing or do not fit optimally into the HOG pathway (O'Rourke and Herskowitz, 1998). The induction of the *FUS-lacZ* reporter upon osmotic shock doubled in the WiPbs2-expressing cells as compared to the cells with ScPbs2 and the WT cells (Figure 2E, *WiPBS2, ScPBS2*, WT). These data suggested that in this *S. cerevisiae* system, WiPbs2 was not optimally activated by the SHO1 branch of the pathway. This is most likely due to inefficient tethering or activation by the ScSho1 osmosensor or the ScSte11 MAPKKK, respectively.

### Characterization of putative osmosensor WiSho1 from *W. ichthyophaga*

**Figure 3:**
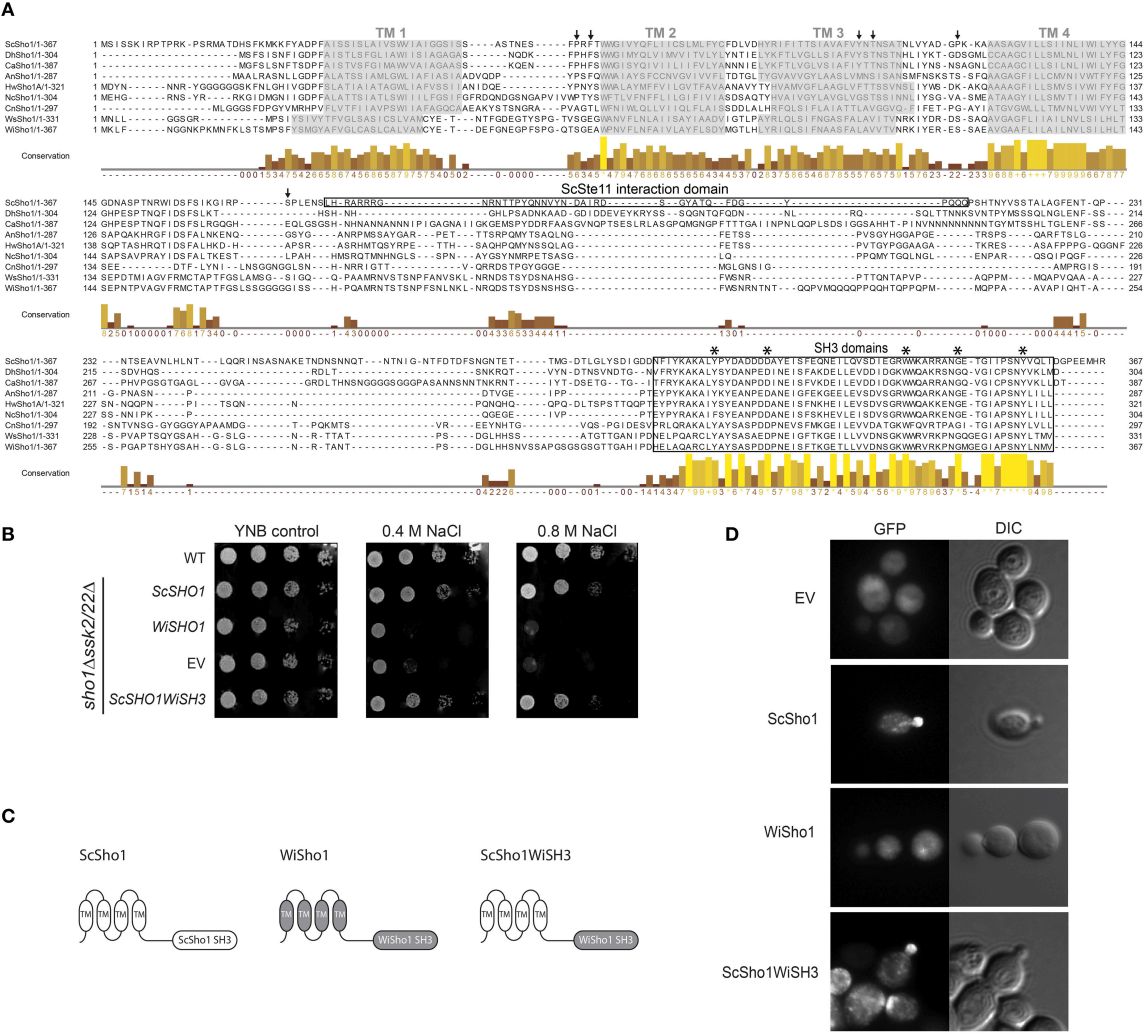
**Bioinformatics and functional analysis of WiSho1**. **(A)** Protein alignment of selected orthologous Sho1 proteins. Prefixes indicate the source organism of Sho1, as in Figure 2A. Gray boxes, putative transmembrane helices (TM1-4); black arrows, ScSho1 residues important for interactions with mucines Hkr1 and Msb2 (Pro63, Phe65, Tyr106, Thr108, Pro120) or for monomerisation (Ser166); framed boxes, ScSte11 interaction domain and SH3 domains; asterisks, important residues for ScSho1 SH3 domain function. The columns demonstrate conservation of amino acids (higher and lighter, greater conservation). See Supplemental Table S3 for the GenBank accession numbers. **(B)** Representative halotolerance rescue assay. The W303 wild-type strain and *sho1*Δ*ssk2/22*Δ strain were transformed as the EV positive control (WT) and *sho1*Δ*ssk2/22*Δ negative control (EV), respectively, and *sho1*Δ*ssk2/22*Δ with *ScSHO1* (positive control), *WiSHO1*, or the *ScSHO1WiSH3* hybrid. **(C)** Cartoons of ScSho1, WiSho1, and the ScSho1WiSH3 hybrid. **(D)** Representative fluorescence microscopy images (GFP) and differential interference contrast (DIC) of *sho1*Δ*ssk2/22*Δ cells transformed as vector alone (EV, negative control) or for GFP-tagged ScSho1, WiSho1, and ScSho1WiSH3.

To investigate the differences in the architectures of the SHO1-branch components in *W. ichthyophaga*, the WiSho1 protein was analyzed. WiSho1 (GenBank accession number KUR62632) is a 367-residue-long protein with a theoretical pI of 6.86 and a molecular weight of 39.22 kDa. This was deduced from the 1104-bp CDS, which is the product of the 1148-bp gene that includes one intron (44 bp long, between nucleotides 406 and 449). The N-terminal part of this WiSho1 protein contains four transmembrane helices and a characteristic SH3 domain (CDD accession number cd11855) at the C-terminus (Figure 3A). In ScSho1, the residues Pro63, Phe65, Tyr106, Thr108, and Pro120 are important for interactions with the putative osmosensing mucines ScHkr1 and ScMsb2 (Tatebayashi et al., 2007). The alterations of these residues resulted in either constitutive signaling activation (for Pro120 variants) or defective signaling (for Pro63, Phe65, Tyr106, and Thr108 variants). However, these residues are not conserved in WiSho1 (Figure 3A, black arrows). In *S. cerevisiae*, Hog1 phosphorylation of ScSho1 at Ser166 (Figure 3A, black arrow) causes its monomerization and regulates the pathway in a negative-feedback manner (Hao et al., 2007). Although this position is not conserved in WiSho1, there are other Ser residues in this region (i.e., original WiSho1 positions 164, 166, 167, 174, 175), which might serve as phosphorylation sites (Figure 3A). Also, the region that is important for interactions with ScSte11 (residues 172–211; Zarrinpar et al., 2004), is not conserved in the Sho1 orthologs used in our alignment (Figure 3A, Ste11 interaction domain). In contrast, the SH3 domain located at the C-terminus is highly conserved in WiSho1 and in the aligned Sho1-like proteins. Interestingly, there is an insertion of Gly that is only seen for the Sho1 SH3 domains of *W. ichthyophaga* and *W. sebi* (Figure 3A, SH3 domains).

To determine whether WiSho1 can complement the function of ScSho1, we expressed the WiSho1 protein in *sho1*Δ*ssk2/22*Δ cells. The data presented in Figure 3B show that the cells that expressed WiSho1 did not grow in the presence of NaCl (Figure 3B, *WiSHO1*, 0.4 M, 0.8 M NaCl). The growth of these cells was reduced to the same level as the EV cells (Figure 3B, EV).

We hypothesized that the SH3 domain of WiSho1 is functional. Therefore we constructed a chimera of the ScSho1 and WiSho1 proteins. We constructed this ScSho1WiSH3 hybrid protein (Figure 3C, ScSho1WiSH3, right) by replacing the SH3 domain of ScSho1 (Figure 3C, ScSho1 SH3, left) with the WiSho1 SH3 domain (Figure 3C, WiSho1 SH3, middle). Indeed, expression of the hybrid ScSho1WiSH3 protein (Figure 3B, *ScSHO1WiSH3*) recovered the osmosensitivity of these *sho1*Δ*ssk2/22*Δ cells. This result confirmed that the WiSho1 SH3 domain is functional when it is integrated into ScSho1.

The potential reason for the osmosensitivity of the WiSho1-expressing *S. cerevisiae* cells is that WiSho1 does not undergo trafficking to the yeast plasma membrane. To investigate this, we tagged the ScSho1, WiSho1, and ScSho1WiSh3 proteins with GFP and analyzed their localizations under fluorescent microscopy. The fluorescence of the WiSho1-GFP was distributed equally within the cell, and resembled that of the EV cells expressing only the GFP tag (Figure 3D, WiSho1, EV). This indicated either false folding of WiSho1-GFP or cleavage of the GFP from the membrane protein. However, the ScSho1WiSH3-GFP hybrid protein was localized predominantly in the plasma membrane at places of polarized growth (Figure 3D, ScSho1WiSH3), which was similar to ScSho1-GFP (used as the positive control; Figure 3D, ScSho1). In conclusion, this functional complementation and appropriate localization of the ScSho1WiSH3 hybrid protein indicates that although full-length WiSho1 did not complement the function of ScSho1 due to its incorrect localization, when the conserved SH3 domain from WiSho1 was inserted into the ScSho1WiSH3 hybrid protein, the HOG activation signal was transmitted to ScPbs2.

### Lower affinity between WiPbs2 and ScSho1WiSH3 results in lower osmotolerance

**Figure 4:**
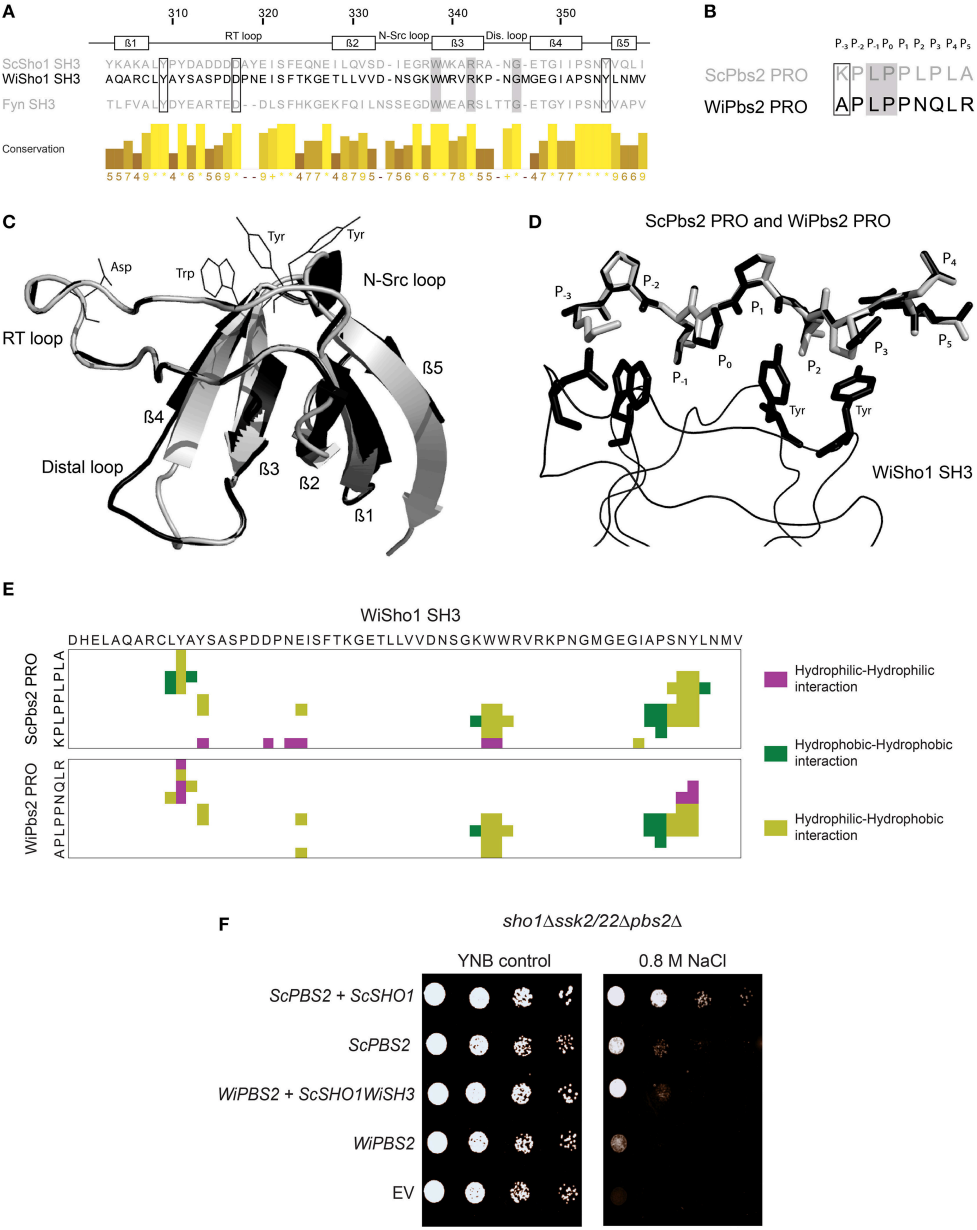
**Interaction between WiSho1 and WiPbs2**. **(A)** Alignment of SH3 domains of ScSho1, WiSho1, and the human tyrosine kinase Fyn. β-Sheet and loop regions are denoted above. Framed and gray boxes, important residues for function of the Sho1 SH3 domains (Marles et al., 2004; Tatebayashi et al., 2006). The columns demonstrate conservation of amino acids (higher and lighter, greater conservation). **(B)** Alignment of proline-rich motifs (PRO) of ScPbs2 and WiPbs2, indicating sites of binding into the SH3 domain pocket (P_−3_–P_5_). Framed and gray boxes, important sites for interactions and signal transduction, respectively. **(C)** Model of superposition of ScSho1 SH3 structure (gray) and WiSho1 SH3 (black). Positions of conserved Asp, Trp, and two Tyr residues of the WiSho1 SH3 domain that are important in the ScSho1 SH3 domain for proline-rich motif binding are shown with black lines. **(D)** Model of WiSho1 SH3 (black ribbon) interaction with ScPbs2 PRO (gray sticks) and the superimposed WiPbs2 PRO (black sticks). Sites of binding are indicated as P_−3_–P_5_ and the important Asp, Trp, and two Tyr residues in WiSho1 SH3 are highlighted (black sticks). **(E)** COCOMAPS property maps, which show the hydrophilic-hydrophilic (purple), hydrophobic-hydrophobic (dark green), and hydrophilic-hydrophobic (olive) residue interactions in the 7 Å atom-to-atom distance from the model of WiSho1 SH3 interaction with ScPbs2 PRO (upper panel) and WiPbs2 PRO (lower panel). **(F)** Representative halotolerance rescue assay. The *sho1*Δ*ssk2/22*Δ*pbs2*Δ strain was transformed as the negative control (EV) and with *WiPBS2* or *ScPBS2*, and *ScSHO1, WiSHO1*, or *ScSHO1WiSH3*.

As the SH3 domain from WiSho1 can interact with ScPbs2, we investigated whether the WiSho1 SH3 domain can also bind to WiPbs2 kinase. We first performed *in-silico* analysis of the interactions between the WiPbs2 proline-rich motif and the WiSho1 SH3 domain. WiPbs2 was submitted to examination with the Eukaryotic Linear Motif Resource (Dinkel et al., 2012) for identification of proline-rich motifs. Multiple PXXP motifs were identified in the N-terminal part of WiPbs2; however, only the APLPPNQLR peptide (i.e., residues 117–125; *E*-value, 1.237e^−03^) met the criteria for being recognized by class I SH3 domains (CDD accession number pfam00018; Saksela and Permi, 2012). Figure 4A shows the alignment of the ScSho1 SH3 domain, the WiSho1 SH3 domain, and the Fyn kinase SH3 domain (Figure 4A, ScSho1 SH3, WiSho1 SH3, Fyn SH3), which highlights the residues that are important for interactions with the proline-rich motif. Alterations of the Tyr309 and Asp317 residues in the RT loop, and the Tyr355 residue, have been reported to lower the binding affinity between the ScSho1 SH3 domain and the ScPbs2 proline-rich motif (Marles et al., 2004; Figure 4A, black framed boxes). Moreover, Trp338 is essential for proline-rich motif binding, while Arg342 and Gly346 replacement causes constitutive activation of the HOG pathway with these altered ScSho1 proteins (Tatebayashi et al., 2006; Figure 4A, gray boxes). All of these residues are well conserved in the WiSho1 SH3 domain. However, in the alignment of the WiPbs2 and ScPbs2 proline-rich motifs shown in Figure 4B (ScPbs2 PRO, WiPbs2 PRO), although the Leu95 and Pro96 of ScPbs2 that have been indicated as essential for signal transduction (Figure 4B, gray-boxed; Maeda et al., 1995; Tatebayashi et al., 2003) are conserved in WiPbs2, the Lys93 of ScPbs2 that has been indicated as important for mediating interactions with acidic residues from SH3 domain (Wu et al., 1995; Marles et al., 2004) is replaced by alanine in WiPbs2.

To further investigate the similarities between these proteins, we modeled the interactions of the WiSho1 SH3 domain and the WiPbs2 proline-rich motif with the SWISS-MODEL software, based on the crystal structure of the ScSho1 SH3 domain complexed with the ScPbs2 proline-rich motif (PDB: 2VKN). The best model that showed reliable GMQE and QMEAN4 values (Biasini et al., 2014) of 0.78 and −1.85, respectively, is illustrated in Figures 4C,D. Superposition of the WiSho1 SH3 domain model on the ScSho1 SH3 domain from the 2VKN structure in Figure 4C shows good alignment. The only important differences detected for this WiSho1 SH3 domain were shorter β4 and β5 sheets, a longer β2 sheet, and a larger distal loop. However, these differences did not influence the positions of the side chains of the conserved residues in the SH3 domains (Figure 4A) that are important for interactions with the proline-rich motif (Figure 4C, Asp, Trp, two Tyr residues, indicated with black lines).

Figure 4D shows the model of the interaction of the proline-rich motifs from ScPbs2 (Figure 4D, gray sticks) and WiPbs2 (Figure 4D, black sticks) with the WiSho1 SH3 domain (Figure 4D, black ribbon). The conserved homologous residues in the SH3 domain that are important for the interactions with the proline-rich motif are also indicated in Figure 4D by black sticks (i.e., WiSho1 SH3, Asp, Trp, two Tyr residues). The WiPbs2 APLPPNQLR proline-rich motif fits well to the major part of the SH3 hydrophobic surface (Figure 4D, P_−2_–P_2_). However, in the WiPbs2 motif, there are the Asn and Gln residues at positions P_2_ and P_3_ respectively, whereas in the ScPbs2 motif these are Lys and Pro (Figures 4B,D). As these Lys and Pro residues from ScPbs2 form hydrophobic interactions with the rings of two Tyr residues at the end of the SH3 hydrophobic surface (Wu et al., 1995; Figure 4D), the Asn and Gln might result in a lower affinity for the binding of the WiPbs2 proline-rich motif to the WiSho1 SH3 domain. Moreover, there is a missing interaction with the acidic Asp residue of the WiSho1 SH3 domain due to the Ala residue at position P_−3_ in the WiPbs2 proline-rich motif (Figure 4D) that might further lower this affinity. However, our estimation of lower affinity only considers the literature-reported residues that are important for interactions between the of SH3 domain and the ScPbs2 proline-rich motif (Wu et al., 1995). We therefore used the COCOMAPS software (Vangone et al., 2011) to cover all possible residue interactions at a 7 Å atom-to-atom distance. In the property maps shown in Figure 4E, the software reports 377 Å^2^ of interaction area, which is formed by nine residues in the ScPbs2 proline-rich motif that are involved in 44 interactions with 18 residues of the WiSho1 SH3 domain (Figure 4E, upper panel). On the other hand, for WiPbs2 PRO-WiSho1 SH3 model, the interaction area of 337 Å^2^ was calculated, as formed by nine residues of the WiPbs2 proline-rich motif that make 38 interactions with 14 residues of WiSho1 SH3 domain (Figure 4E, lower panel). Taking these *in-silico* data together, they predict that the interaction of the proline-rich motif from WiPbs2 with the WiSho1 SH3 domain will be weaker than that between the ScPbs2 proline-rich motif and the WiSho1 SH3 domain.

To determine whether this predicted weaker interaction of the WiPbs2 and the WiSho1 SH3 domain is also reflected in lower osmotolerance of *S. cerevisiae*, in the *sho1*Δ*ssk2/22*Δ*pbs2*Δ cells we simultaneously expressed the kinase WiPbs2 and ScSho1WiSH3 (Figure 4F). Indeed, the growth of these WiPbs2-expressing and ScSho1WiSH3-expressing cells was reduced in comparison to the positive-control cells expressing ScPbs2 and ScSho1 (Figure 4F, *WiPBS2* + *ScSHO1WiSH3, ScPBS2* + *ScSHO1*), and similar to the cells expressing only the ScPbs2 kinase (Figure 4F, *ScPBS2*). The growth of the cells expressing only WiPbs2 (Figure 4F, *WiPBS2*) was even more reduced, and in the case of the EV (Figure 4F, EV; negative control), the growth was completely inhibited. This supports the results of the *in-silico* prediction that the WiSho1 SH3 domain binds WiPbs2 with lower affinity.

### Insertion of the ScPbs2 proline-rich motif into WiPbs2 results in stronger interactions with ScSho1WiSH3

**Figure 5:**
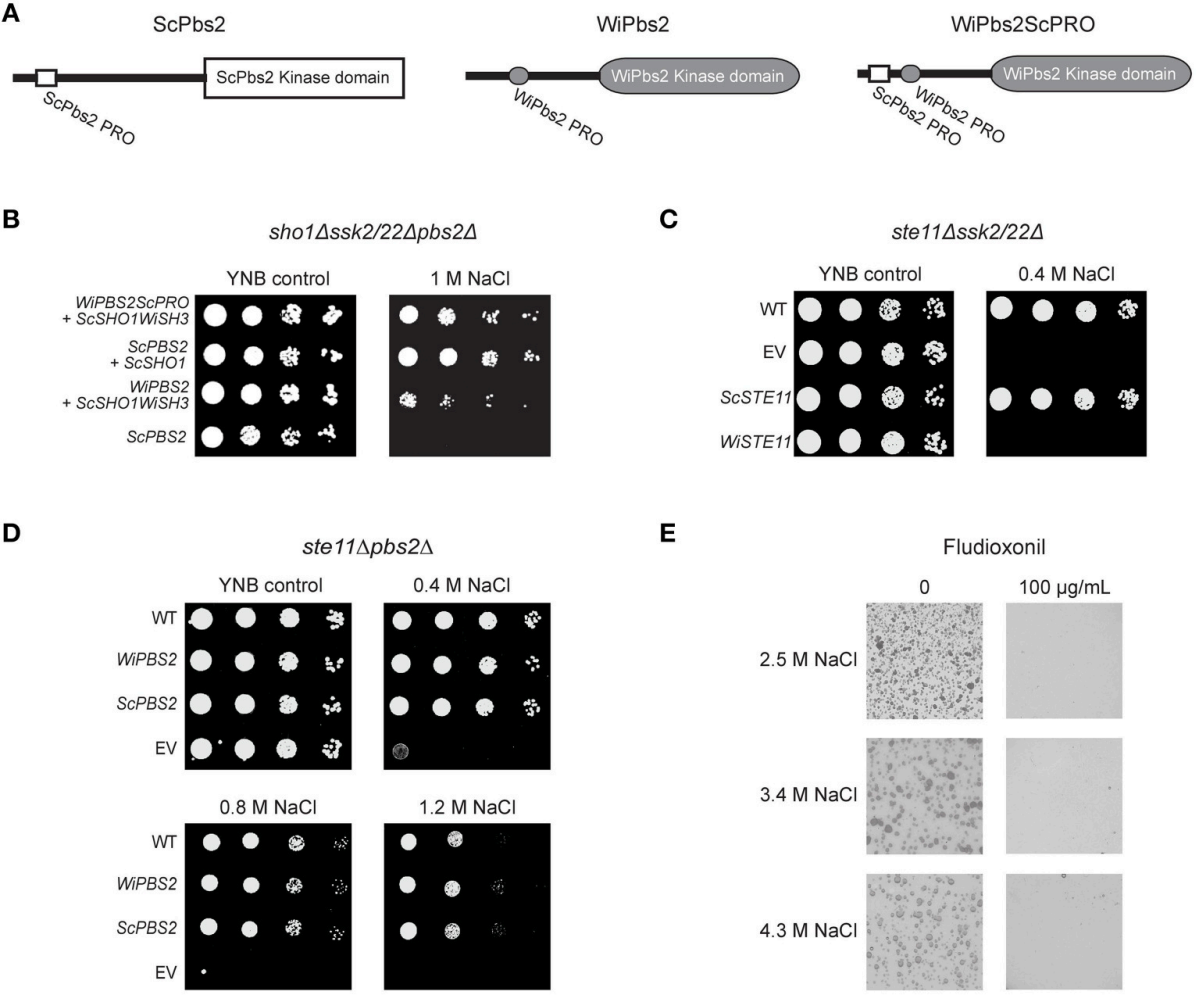
**Expression of WiPbs2 with inserted ScPbs2 proline-rich motif**. **(A)** Schematic representation of ScPbs2, WiPbs2, and WiPbs2ScPRO. The proline-rich motif VNKPLPPLPVAG from ScPbs2 was inserted into the N-terminus of WiPbs2 to obtain the WiPbs2ScPRO altered protein. **(B–D)** Representative halotolerance rescue assays. **(B)** The *sho1*Δ*ssk2/22*Δ*pbs2*Δ strain was transformed with the negative control (*ScPBS2* + EV) and the positive control (*ScPBS2* + *ScSHO1*), and with *ScSHO1WiSH3* and *WiPBS2ScPRO* or *WiPBS2*. **(C)** The Y00000 wild-type strain and *ste11*Δ*ssk2/22*Δ strain were transformed as the EV positive control (WT) and *ste11*Δ*ssk2/22*Δ negative control (EV), respectively, and *ste11*Δ*ssk2/22*Δ with *ScSTE11* (positive control) or *WiSTE11*. **(D)** The W303 wild-type strain and *ste11*Δ*pbs2*Δ strain were transformed as the EV positive control (WT) and *ste11*Δ*pbs2*Δ negative control (EV), respectively, and *ste11*Δ*pbs2*Δ with *ScPBS2* (positive control) or *WiPBS2*. **(E)** Representative plates of growth of *W. ichthyophaga* in the absence (left) and presence (right) of 100 μg/mL fludioxonil, on plates containing 1.7, 3.4, and 5.1 M NaCl.

Upon SHO1 branch activation in *S. cerevisiae*, ScSho1 recruits ScPbs2 to the membrane, where it is activated via phosphorylation by the MAPKKK ScSte11. We additionally investigated whether inefficient activation by ScSte11 kinase, rather than weaker binding with the WiSho1 SH3 domain, was the reason for the lower osmotolerance of these cells expressing WiPbs2 and ScSho1WiSH3 (Figure 4F, *WiPBS2* + *ScSHO1WiSH3*). Using oligonucleotide-directed mutagenesis, we constructed an altered WiPbs2 protein (Figure 5A, WiPbs2ScPRO), which contained an artificial insertion of the proline-rich motif VNKPLPPLPVAG from ScPbs2 (Figure 5A, ScPbs2) in the N-terminal portion of WiPbs2 (Figure 5A, WiPbs2). When this WiPbs2ScPRO mutant protein was expressed simultaneously with the ScSho1WiSH3 protein in the *sho1*Δ*ssk2/22*Δ*pbs2*Δ cells, the osmotolerance of these cells was enhanced on 1 M NaCl in comparison to the cells expressing WiPbs2 and ScSho1WiSH3 (Figure 5B, *WiPBS2ScPRO* + *ScSHO1WiSH3, WiPBS2* + *ScSHO1WiSH3*). This indicates that the insertion of the ScPbs2 proline-rich motif into WiPbs2 leads to stronger interactions with ScSho1WiSH3, and consequently to more efficient pathway activation and greater osmotolerance. Moreover, this also suggests that the interaction between WiPbs2 and the WiSho1 SH3 domain, rather than WiPbs2 and ScSte11, is the limiting step in WiPbs2 activation via the SHO1 branch of the HOG pathway.

However, despite this indication of efficient WiPbs2 activation by ScSte11 in *S. cerevisiae*, the expression of the *W. ichthyophaga* MAPKKK WiSte11 in *ste11*Δ*ssk2/22*Δ cells did not abolish the osmosensitivity of these cells (Figure 5C, *WiSTE11*). Instead, their growth was completely inhibited already at the low 0.4 M NaCl concentration (Figure 5C, *WiSTE11*). In contrast, the ScSte11-expressing cells (Figure 5C, *ScSTE1*; the positive control) were as osmoresistant as the WT cells (Figure 5C, *ScSTE1*, WT). Although the WiSte11 kinase contains the conserved N-terminal Sterile alpha motif and C-terminal Kinase domain (Supplemental Figure S2), the highly conserved phosphorylation sites for Ste20 (van Drogen et al., 2000; Supplemental Figure S2, Ser281, Ser285, Thr286), and the moderately conserved Sho1 binding region (Tatebayashi et al., 2006; Supplemental Figure S2), it is 422 amino acids larger than ScSte11 (Figure 1). This might be the reason for this inefficient functional complementation that results in high osmosensitivity of the WiSte11-expressing *ste11*Δ*ssk2/22*Δ cells.

### Conserved ScSsk2/22-WiPbs2 interaction and fludioxonil sensitivity indicate the importance of the phosphorelay system in *W. ichthyophaga*

As the growth of the *pbs2*Δ cells expressing either WiPbs2 or ScPbs2 was identical (Figure 2C), these two kinases likely have similar roles in the SLN1 branch, which masks the less efficient activation of WiPbs2 by the SHO1 branch (Figures 2D, 4F). To confirm this, we expressed WiPbs2 in the *ste11*Δ*pbs2*Δ cells, where only the two-component SLN1 branch was functional. Indeed, these data show that the growth of these WiPbs2-expressing cells is identical to the ScPbs2-expressing cells at all NaCl concentrations used (Figure 5D). This indiscriminate activation of WiPbs2 and the ScPbs2 kinases by the ScSsk2/ScSsk22 MAPKKK in *S. cerevisiae* indicates that interactions in this part of the pathway are evolutionarily highly conserved.

In contrast to *S. cerevisiae*, which has the two paralogs ScSsk2 and ScSsk22, the genome of *W. ichthyophaga* contains only one ortholog, WiSsk2. This is a 1357-amino-acid protein with a conserved kinase domain at its C terminus (Supplemental Figure S3). The *S. cerevisiae* ScSsk2 N-terminus contains the ScSsk1 binding domain (Posas et al., 1998; Supplemental Figure S3) and the activation domain (Horie et al., 2008; Supplemental Figure S3), which is also known as the autoinhibitory domain (Tatebayashi et al., 2003). Although the N-terminal sequence of WiSsk2 is not highly conserved (Supplemental Figure S3), the data in Figure 5D show that in *S. cerevisiae* the WiPbs2 kinase is preferentially activated via ScSsk2/ScSsk22 of the SLN1 branch.

The SLN1 branch in *S. cerevisiae* is controlled by the transmembrane histidine kinase ScSln1. *W. ichthyophaga* does not have the ScSln1 ortholog. Possibly, instead, the cytosolic Group III histidine kinase WiNik1 is involved in osmosensing and fludioxonil sensitivity, as has been shown for *C. neoformans* strains with constitutive CnHog1 phosphorylation (Bahn et al., 2007). *W. ichthyophaga* also shows constitutive WiHog1 phosphorylation under noninducing conditions (Konte and Plemenitaš, 2013), and this provides the prerequisite to test the sensitivity of cells to fludioxonil. In the presence of fludioxonil, the growth of *W. ichthyophaga* cells was completely inhibited at all NaCl concentrations used (Figure 5E).

### Cells expressing WiHog1A have greater osmosensitivity and show greater cross-talk

**Figure 6:**
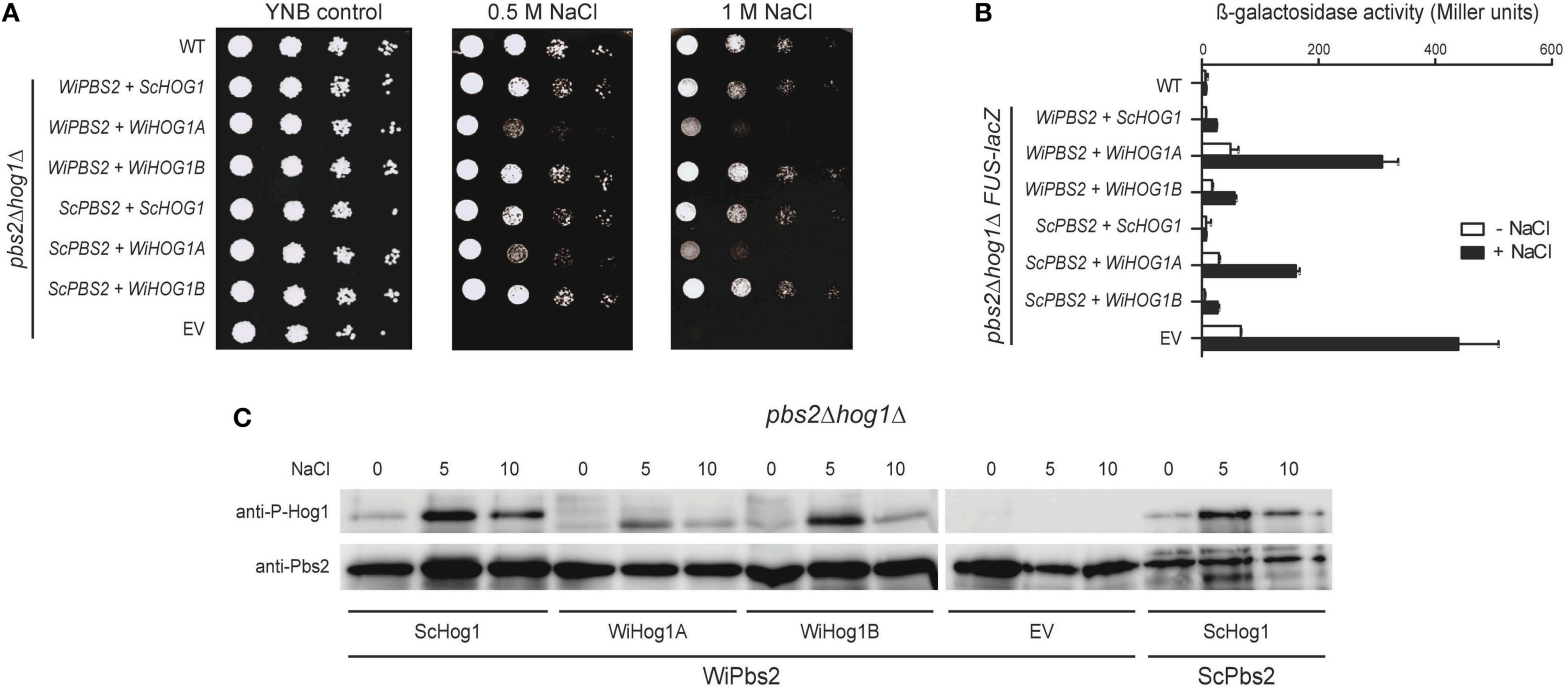
**Simultaneous expression of WiPbs2, and WiHog1A or WiHog1B in ***S. cerevisiae pbs2***Δ***hog1***Δ cells**. **(A)** Halotolerance rescue assay. The S1278b WT strain and *pbs2*Δ*hog1*Δ strain were transformed as the EV positive control (WT) and *pbs2*Δ*hog1*Δ negative control (EV), respectively, and *pbs2*Δ*hog1*Δ with the indicated combinations of *WiPBS2* or *ScPBS2*, and *ScHOG1, WiHOG1A*, or *WiHOG1B*. **(B)** Cross-talk activation of the mating pathway, as β-galactosidase activity, in S1278b*FUS1lacZ* and *pbs2*Δ*hog1*Δ*FUS-lacZ* cells transformed as in **(A)**. Activities were measured before and after 1 M NaCl for 4 h. Data are means ± SD (*n* = 3). **(C)** Western blotting of *pbs2*Δ*hog1*Δ cells expressing WiPbs2 and ScHog1, WiHog1A, or WiHog1B. Expression of ScPbs2 and ScHog1 was the positive control, and of WiPbs2 alone (EV) was the negative control. Expressed Pbs2 and phosphorylated Hog1 kinases were immunodetected in lysates before and after (5, 10 min) application of 0.8 M NaCl. The proteins were immunodetected on two separate membranes as indicated by the spliced image.

When expressed in *S. cerevisiae*, WiHog1A only partially rescued the osmosensitive phenotype of *hog1*Δ cells (Konte and Plemenitaš, 2013). One of the possible reasons for this poor performance of WiHog1A in *S. cerevisiae hog1*Δ cells might be weaker activation by ScPbs2, which differs from WiPbs2 in its sequence, and is not the native activating kinase of WiHog1A. To address this, in *S. cerevisiae pbs2*Δ*hog1*Δ cells, we expressed the MAPKKs WiPbs2 or ScPbs2 together with the MAPKs WiHog1A, WiHog1B, or ScHog1. In this way, we tested whether the salt-tolerance of the *pbs2*Δ*hog1*Δ cells was improved after activation of the WiHog1 MAPKs by their native activating MAPKK, WiPbs2. The growth of the cells expressing both WiPbs2 and WiHog1A was reduced to the same level as the growth of the cells expressing ScPbs2 and WiHog1A (i.e., used as the control), when compared to the growth of the cells expressing either of the two Pbs2 MAPKKs (i.e., WiPbs2 or ScPbs2) together with WiHog1B or ScHog1 (Figure 6A, 0.5 M NaCl, 1 M NaCl). This is in agreement with our previous data in the *S. cerevisiae hog1*Δ cells, where only WiHog1B, and not WiHog1A, fully complemented the function of ScHog1 (Konte and Plemenitaš, 2013).

To further confirm these data, we performed β-galactosidase assays (Miller, 1972) with the *FUS-lacZ* reporter. We integrated the *FUS-lacZ* reporter system into the genome of the *S. cerevisiae pbs2*Δ*hog1*Δ and S1278b WT cells prior to transformation with WiPbs2 or ScPbs2, and WiHog1A, WiHog1B, or ScHog1. Figure 6B shows the results for the activation of this mating pathway upon 1 M NaCl shock, for 4 h. As expected, the cells with EV (Figure 6B, EV), which were the positive control, showed the greatest β-galactosidase activity (i.e., the highest cross-talk), while the WT cells (Figure 6B, WT) and the cells expressing ScPbs2 and ScHog1 (Figure 6B, *ScPBS2*+*ScHOG1*), both as negative controls, showed almost no activation when stressed with NaCl. In the cells expressing ScHog1 or WiHog1B, we detected low β-galactosidase activity regardless of the co-expressed MAPKKs ScPbs2 or WiPbs2 (Figure 6B, *ScPBS2*+*WiHOG1B, WiPBS2*+*ScHOG1, WiPBS2*+*WiHOG1B*). On the other hand, when WiHog1A was expressed simultaneously with ScPbs2, the induction of β-galactosidase reached more than 150 Miller units, and even doubled to 300 units when it was co-expressed with WiPbs2 (Figure 6B; *ScPBS2*+*WiHOG1A, WiPBS2*+*WiHOG1A*).

Finally, we directly determined the levels of activation of the Hog1 MAPKs by WiPbs2, to determine whether the reason for the poor performance of the cells expressing WiHog1A and WiPbs2 was a result of differential phosphorylation by WiPbs2. The cells expressing WiPbs2 and one of WiHog1A, WiHog1B, or ScHog1 were shocked with 1 M NaCl. The phosphorylation patterns were similar for the WiHog1A, WiHog1B, and ScHog1 MAPKs. The greatest intensity of phosphorylation was detected after 5 min of the 1 M NaCl shock (Figure 6C, NaCl 5), and decreased after 10 min (Figure 6C, NaCl 10) for all of the MAPKs, regardless of the MAPKK expressed (Figure 6C; WiPbs2, ScPbs2). This high phosphorylation after 5 min of the 1 M NaCl shock suggests that WiPbs2 efficiently activates WiHog1A, WiHog1B, and ScHog1, with slightly lower intensity in the case of WiHog1A.

## Discussion

The obligate halophilic fungus *W. ichthyophaga* grows in media enriched with NaCl concentrations from 1.7 to 5.1 M (Zajc et al., 2014). This is what makes *W. ichthyophaga* an interesting model organism for investigating the mechanisms of osmotolerance and osmosensing. Herein, we identified components of the HOG pathway, which is the central osmosensing pathway of *W. ichthyophaga* (Konte and Plemenitaš, 2013). We analyzed the genome of *W. ichthyophaga*, heterologously expressed the selected *W. ichthyophaga* HOG signaling proteins, and studied their functions and interactions to further illucidate the architecture of the HOG pathway in this halophilic basidiomycetous fungus.

*S. cerevisiae* has previously been used as a system for expression of mammalian MAP kinases, which in yeast lose their ability to function normally and become spontaneously phospohorylated (Levin-Salomon et al., 2009). The turgor sensor function was identified in *Arabidopsis thaliana* as the plant cytokinin receptor 1 (Cre1) when it replaced the function of ScSln1 histidine kinase at high osmolarity (Reiser et al., 2003), and in a similar manner, *A. thaliana* ATMEKK1 kinase replaced the function of ScSte11 (Covic et al., 1999). The *Cicer arietinum* zinc finger protein CaZF was even shown to enhance the osmotolerance of yeast, with effective accumulation of glycerol and induction of stress genes in a HOG-dependant and calcineurin-dependant manner (Jain et al., 2009). *S. cerevisiae* has also been used for the characterization of many fungal kinases, including the Hog1-like kinases from *Zygosaccharomyces rouxii* (Iwaki et al., 1999) and the DhPbs2 kinase from *Debaryomyces hansenii* (Bansal et al., 2001), and recently also for the molecular dissections of the *D. hansenii* DhNik1 (Furukawa et al., 2012) and *C. albicans* CaNik1 (El-Mowafy et al., 2013) histidine kinases. As a step further, in our investigation, we comprehensively reconstructed the pathway with the heterologous expression of the core module of the pathway, which consists of the MAPKK WiPbs2 and its interacting proteins, WiSho1, WiSte11, and WiHog1A and B. Moreover, we confirmed the significance of the two-component HOG-pathway system in *W. ichthyophaga* cells, using the fludioxonil sensitivity assay.

It has been reported recently that within the *S. cerevisiae* SHO1 branch there are two signaling sub-branches that can be distinguished, known as HKR1 and MSB2 (Tanaka et al., 2014). ScSho1 acts as a true osmosensor only in the HKR1 sub-branch, where upon hyperosmolar stimulus, the ScOpy2-ScSte50 complex recruits ScSte11, the ScHkr1, and ScCdc42 proteins recruit ScSte20/ScCla4, and ScSho1 recruits ScPbs2 to the membrane. ScSho1 also forms a multi-component signaling complex with ScOpy2 and ScHkr1, and the high-osmolarity-dependent structural changes in the ScSho1 transmembrane domains induce its binding with ScSte50 (Tatebayashi et al., 2015). In the genome of *W. ichthyophaga*, we were not able to identify a considerable number of the orthologs that are involved in the SHO1 branch of the osmosensing apparatus in *S. cerevisiae*; namely, the mucins Msb2 and Hkr1, and the membrane anchor Opy2. However, other orthologs of this branch are present, with significant size expansion in the case of the kinases WiSte50, WiSte20, and WiSte11. The important interactions for the activation of ScPbs2 by the SHO1 branch also include ScSho1 binding with the ScPbs2 proline-rich motif (Marles et al., 2004) and docking of ScSte11 (Tatebayashi et al., 2006). Although these interaction motifs are poorly conserved in the sequence of WiPbs2, there is an APLPPNQLR motif in the N-terminal part of WiPbs2 (residues 117–125) that represents the only putative proline-rich motif that can be recognized by the class I SH3 domains.

We compared the binding of both ScPbs2 and the WiPbs2 proline-rich motif with the WiSho1 SH3 domain from *W. ichthyophaga*. The analysis of the model with COCOMAPS predicted weaker interactions for the binding of the WiPbs2 proline-rich motif and the WiSho1 SH3 domain. This prediction of weaker interactions was shown by the lower osmotolerance and the greater cross-talk activation in WiPbs2-expressing *ssk2/22*Δ*pbs2*Δ cells, and this was additionally confirmed by the lower osmotolerance of the *sho1ssk2/22*Δ*pbs2*Δ cells that expressed WiPbs2 and the ScSho1WiSH3 hybrid protein. As the insertion of the ScPbs2 proline-rich motif into the N-terminus of WiPbs2 increased the osmotolerance of *S. cerevisiae* cells, it is possible that the weaker binding of WiPbs2 and ScSho1WiSH3 was the cause of the weak activation of WiPbs2 via the SHO1 branch. This assumption was further supported when the insertion of the ScPbs2 proline-rich motif into the N-terminal part of WiPbs2 increased the osmotolerance of *S. cerevisiae* cells, most probably by strengthening the binding of the hybrid protein WiPbs2ScPRO to the ScSho1WiSH3 hybrid osmosensor. Although these data also showed that WiPbs2 can be efficiently activated by ScSte11, the WiSte11-expressing *S. cerevisiae ste11*Δ*ssk2/22*Δ cells were osmosensitive, which indicated that WiSte11 cannot complement the function of ScSte11.

**Figure 7:**
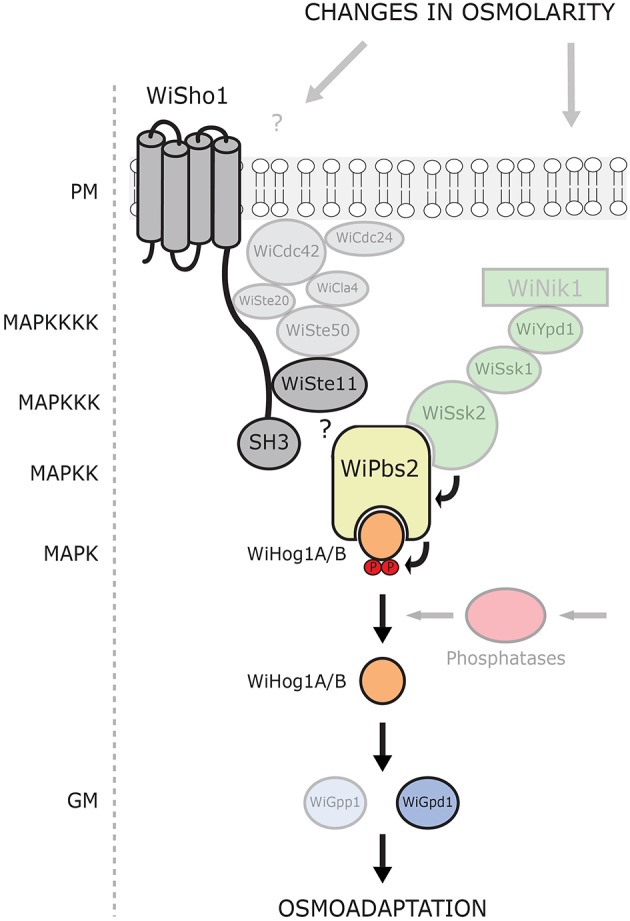
**Model of current understanding of the HOG pathway architecture in ***W. ichthyophaga*****. Putative HOG-pathway components identified in the genome of *W. ichthyophaga* are shown. Proteins experimentally characterized to date are in bold. The components of the SHO1-like branch are shown in gray, and the two-component (SLN1-like) branch in green. The MAPK hierarchy is indicated (left). PM, plasma membrane; SH3, Src homology 3 domain; P, phosphate groups; GM, glycerol metabolism.

This, and the poor interactions between the WiSho1 SH3 domain and the WiPbs2 proline-rich motif that we have shown here, combined with the demonstration that the glycosylated transmembrane orthologs Hkr1, Msb2, and Opy2 (the essential components of osmosensing in *S. cerevisiae*; Tatebayashi et al., 2007) were not found in *W. ichthyophaga*, suggest that the SHO1-branch is not involved in HOG signaling in *W. ichthyophaga* (Figure 7). This is also in line with the results reported for *A. nidulans* PbsA (AnPbs2; Furukawa et al., 2005). When expressed in *S. cerevisiae*, AnPbs2 is not activated by ScSho1 in *ssk2/22*Δ*pbs2*Δ cells, whereas ShoA (AnSho1) is functional in the *sho1*Δ*ssk2/22*Δ background (Furukawa et al., 2005). Similarly, the HwSho1 proteins from the extremely halotolerant *H. werneckii* can bind ScPbs2 (Fettich et al., 2011), but an interaction of ScSho1 with HwPbs2 kinases in *S. cerevisiae* was not confirmed (Plemenitaš et al., 2014). Also in *Candida* sp., which is closely related to *S. cerevisiae*, Sho1 only has an indirect role in the osmotic stress response, and it is not involved in osmosensing (Jandric et al., 2013; Herrero-de-Dios et al., 2014). On the other hand, the SHO1 and SLN1 branches are functional in *Kluyveromyces lactis* (Velazquez-Zavala et al., 2015), which suggests that the existence of the sophisticated SHO1-branch osmotic signal perception in not limited to *S. cerevisiae*. Even though the *W. ichthyophaga* genome does not contain the orthologs of typical osmosensors, it cannot be excluded that some other unconventional osmosensors and membrane anchors are present that can recruit WiSte50 (e.g., by interacting with its Ras-association domain). Hypothetically, WiSte50 would than bind WiSte11 through Sterile alpha motif interactions, and WiPbs2 through the SH3 domains (for details, see the domain structure of WiSte50 in Figure 1), providing the scaffold for the activation of WiPbs2 by WiSte11, without the need for participation of WiSho1.

In contrast to the SHO1 branch, the two-component SLN1 branch orthologs appear to have pivotal roles. Although the Ssk2/Ssk22 binding site of WiPbs2 is not highly conserved, the growth in NaCl of *S. cerevisiae pbs2*Δ and *ste11*Δ*pbs2*Δ cells that express WiPbs2 is similar to those expressing ScPbs2. This suggests that WiPbs2 is efficiently activated by ScSsk2/ScSsk22 kinase in *S. cerevisiae* and has a comparable role to ScPbs2 when activated through the SLN1 branch. All of the orthologs of the *S. cerevisiae* SLN1 branch, except the Sln1 transmembrane histidine kinase, are present in the genome of *W. ichthyophaga* (Figures 1, 7). In *S. cerevisiae*, ScSln1 is the only histidine kinase, but in other fungi there are several groups (Catlett et al., 2003; Lenassi and Plemenitaš, 2007; Ma and Li, 2013) The genome of *W. ichthyophaga* contains four hybrid histidine kinases that belong to groups I or II, III and VIII. It has been shown for several fungi that the Nik1 orthologs from group III are involved in the two-component HOG-pathway signaling (Bahn, 2008; El-Mowafy et al., 2013). We therefore speculate that the HAMP domain repeats in WiNik1 (Figure 1) are involved in osmosensing in *W. ichthyophaga*, as has been demonstrated for *D. hansenii* DhNik1 (Kaur et al., 2014), *C. albicans* CaNik1 (El-Mowafy et al., 2013), and *Candida lusitaniae* ClNik1 (Randhawa et al., 2016). Furthermore, it has been shown that the group III histidine kinases are also involved in fungal sensitivity to fludioxonil, which makes this phenylpyrrole fungicide a potent tool for investigations into group III histidine kinase signaling. Interestingly, in the fludioxonil-sensitive *C. neoformans* serotype A strain H99, CnHog1 is constitutively phosphorylated under normal conditions and dephosphorylated under osmotic shock (Bahn et al., 2007). In *C. neoformans*, the group III histidine kinase Tco1 (CnNik1) is involved in osmosensing, as well as in fludioxonil sensitivity (Bahn et al., 2006). As *W. ichthyophaga* also contains the group III histidine kinase WiNik1 (Figures 1, 7) and shows similar constitutive phosphorylation patterns under optimal (noninducing) conditions (Konte and Plemenitaš, 2013), we investigated the sensitivity of *W. ichthyophaga* cells to fludioxonil. Indeed, the presence of fludioxonil completely inhibited the growth of *W. ichthyophaga* cells at all NaCl concentrations used. This is also in line with the recently published results of Nik1 kinase involvement in the fungicide sensitivity and osmotic signaling in *M. oryzae* (Jacob et al., 2015) and *C. lusitaniae* (Randhawa et al., 2016). As we were not able to produce any HOG pathway knock-out mutants of the halophilic *W. ichthyophaga* even after multiple attempts, this result of fludioxonil sensitivity indicates the importance of the group III histidine kinase signaling and the two-component signal transduction in *W. ichthyophaga*, and suggests the involvement of the HOG pathway in survival of *W. ichthyophaga*, even at its optimal NaCl concentration of 3.4 M.

Unconventional constitutive phosphorylation of Hog1 kinases under nonstress conditions has only been observed in the halophilic *W. ichthyophaga* (Konte and Plemenitaš, 2013) and the pathogenic *C. neoformans* serotype A strain H99 (Bahn et al., 2005) to date. Such constitutive phosphorylation patterns of HOG pathway regulation are the reverse to those of yeast and the majority of other fungi, where Hog1 is dephosphorylated under nonstress conditions and phosphorylated upon osmotic shock (Krantz et al., 2006b; Saito and Posas, 2012; Schmidt-Heydt et al., 2013). Interestingly, when the cells of halotolerant *H. werneckii* are grown in up to 3 M NaCl, the phosphorylation patterns are similar to that of *S. cerevisiae*, but when they are exposed to higher salinities, HwHog1 is phosphorylated constitutively (Kejžar et al., 2015). These hybrid phosphorylation patterns might represent the foundations of *H. werneckii* extreme halotolerance.

However, analysis of the HOG pathway components in the closely related *C. neoformans* strains H99 and JEC21, which show different phosphorylation patterns (constitutive vs. transient), has demonstrated that the change of Phe240 to Leu in the MAPKKK CnSsk2 is the sole reason for constitutive phosphorylation of CnHog1 in the serotype A strain H99 (Bahn et al., 2007). This residue is located in the putative CnSsk1 binding domain on CnSsk2, which most probably changes the mechanism of CnSsk2 regulation by CnSsk1. Interestingly, when compared to other *C. neoformans* strains, serotype A strain H99 is more resistant to various environmental stresses, including osmotic stress, although it is sensitive to fludioxonil (Kojima et al., 2006; Bahn et al., 2007). As the alignment of CnSsk2 and WiSsk2 revealed the presence of a Leu residue at the homologous site in WiSsk2 (Supplemental Figure S3), we propose that this might be the reason for constitutive phosphorylation of WiHog1 also in *W. ichthyophaga*. Furthermore, our data suggest that this constitutive phosphorylation originates from a component upstream of WiHog1 and WiPbs2, and not from their autophosphorylation. This assumption is supported by our finding that when expressed in *S. cerevisiae pbs2*Δ*hog1*Δ cells, WiHog1 and WiPbs2 show normal phosphorylation patterns that are similar to those of ScHog1 and ScPbs2, and can even replace their function.

In a previous study, we showed that the two MAPK paralogs WiHog1A and WiHog1B from *W. ichthyophaga* differ, as WiHog1A did not complement the function of ScHog1 as efficiently as WiHog1B in *S. cerevisiae hog1*Δ cells (Konte and Plemenitaš, 2013). This *HOG1*-like gene duplication is an unusual trait, which has only been identified in *W. sebi* (Padamsee et al., 2012), *H. werneckii* (Kejžar et al., 2015), *Z. rouxii* (Iwaki et al., 1999), *A. nidulans* (Furukawa et al., 2005), and *A. fumigatus* (de Oliveira Bruder Nascimento et al., 2016). While the paralogous Hog1-like kinases from *H. werneckii* and *Z. rouxii* appear to be redundant, in *A. nidulans* and *A. fumigatus* their roles are divergent. The *A. fumigatus* HogA (AfSakA) is essential for osmosensing, while the *A. nidulans* AnSakA is not (Ma and Li, 2013; de Oliveira Bruder Nascimento et al., 2016). On the other hand, in *A. fumigatus*, the paralogous AfMpkC kinase has been linked to the use of polyalcohol sugars (Reyes et al., 2006) and virulence (de Oliveira Bruder Nascimento et al., 2016), while the exact role of *A. nidulans* AnMpkC has not been defined (Furukawa et al., 2005). In the present study we expressed WiHog1A or WiHog1B together with WiPbs2 in *pbs2*Δ*hog1*Δ cells. When challenged with NaCl, the cells expressing WiPbs2 and WiHog1A showed greater cross-talk activation, greater osmosensitivity, and slightly lower WiHog1A phosphorylation, as compared to the cells expressing WiPbs2 and WiHog1B. However, the dephosphorylation of WiHog1A and WiHog1B that occurred 10 min after exposure to NaCl show that both of these MAPKs can efficiently activate *S. cerevisiae* phosphatases for their negative-feedback regulation. While WiHog1A cannot inhibit the cross-talk but efficiently binds to the *GPD1* promoter in *S. cerevisiae*, WiHog1B can inhibit the cross-talk and can also efficiently induce *GPD1-lacZ* (Konte and Plemenitaš, 2013). This, and that the induction of the *WiHOG1B* mRNA transcripts doubled in comparison to *WiHOG1A* transcripts in *W. ichthyophaga* upon hyperosmotic shock (Lenassi et al., 2011; Konte and Plemenitaš, 2013), indicate that WiHog1B is also the primary HOG pathway kinase in *W. ichthyophaga*.

One of the major and first Hog1 targets in *S. cerevisiae* is glycerol transport and metabolism. Upon hyperosmotic shock, the aquaglyceroporin Fps1 is rapidly closed to prevent the efflux of glycerol, and there is strong induction of the expression of glycerol proton symporter Stl, as well as of genes for glycerol synthesis (*GPD1, GPP1*, and *GPP2*; Hohmann et al., 2007). The genome of *W. ichthyophaga* contains three aquaglyceroporins and four putative glycerol Stl1-like transporters (Zajc et al., 2013). Key enzymes involved in glycerol metabolism are also present and conserved in *W. ichthyophaga*. The expression of the gene encoding WiGpd1 was previously shown to be salt-induced (Lenassi et al., 2011), however, the genome of *W. ichthyophaga* also contains another paralogous Gpd enzyme. On the other hand, only the ortholog of Gpp1, WiGpp1, is present, without Gpp2 (Zajc et al., 2013). This salt-responsive *WiGPD1* expression was much lower compared to *HwGPD1* from *H. werneckii* (Lenassi et al., 2011), which might reflect the halophilic vs. halotolerant natures of *W. ichthyophaga* and *H. werneckii*, respectively. Interestingly, in *C. neoformans*, the genes that encode Stl1-like transporter, aquaglyceroporin, Gpd1, and Gpp1 are all salt responsive and induced in a HOG-dependant manner (Ko et al., 2009). Glycerol accumulation in *C. neoformans* is regulated through a Hog1-regulated kinase 1 (Hkr1), which is induced via the two-component system (Kim et al., 2011). It has also been shown that fludioxonil promotes glycerol overaccumulation, which causes cell swelling and death (Kojima et al., 2006). Similarly, glycerol overaccumulation might also be the reason for fludioxonil sensitivity of *W. ichthyophaga* cells.

Taken together, our investigation of the architecture of the *W. ichthyophaga* HOG pathway shows that the interactions of WiPbs2 kinase and the SHO1-branch orthologs are not conserved (Figure 7), which in turn suggests that these orthologs are not involved in the activation of WiPbs2. On the other hand, the ScSsk2/ScSsk22 activation of WiPbs2 is fully conserved, and *W. ichthyophaga* cells are sensitive to fludioxonil, which highlights the importance of the two-component HOG pathway signaling in *W. ichthyophaga* (Figure 7). As demonstrated to be necessary for higher stress-resistance of *C. neoformans*, the constitutive two-component HOG-pathway phosphorylation could also help *W. ichthyophaga* to survive in its naturally hypersaline environments. However, the exact mechanism of the hyperosmotic signal processing and the role of phosphatases in pathway activation by dephosphorylation remains the subject of further studies.

## Author contributions

AP provided funding. AP and TK conceived and designed the study and the experiments. TK performed the experiments. TK, AP, and UT analyzed and interpreted the data. TK wrote the manuscript. AP and UT provided critical revision. All of the authors reviewed the results and approved the final version of the manuscript.

## Funding

This study was funded in part by Research Grant P1-0170, and in part by a Young Researcher Fellowship, from the Slovenian Research Agency.

### Conflict of interest statement

The authors declare that the research was conducted in the absence of any commercial or financial relationships that could be construed as a potential conflict of interest.
