# Supplementary material for: Reconstruction of the High-Osmolarity Glycerol (HOG) Signaling Pathway from the Halophilic Fungus Wallemia ichthyophaga in Saccharomyces cerevisiae
Source: Front Microbiol. 2016 Jun 13;7:901. doi: 10.3389/fmicb.2016.00901 (PMC4904012; doi:10.3389/fmicb.2016.00901)
Supplement: Supplementary file 1 [file Image1.pdf]

Supplementary Material

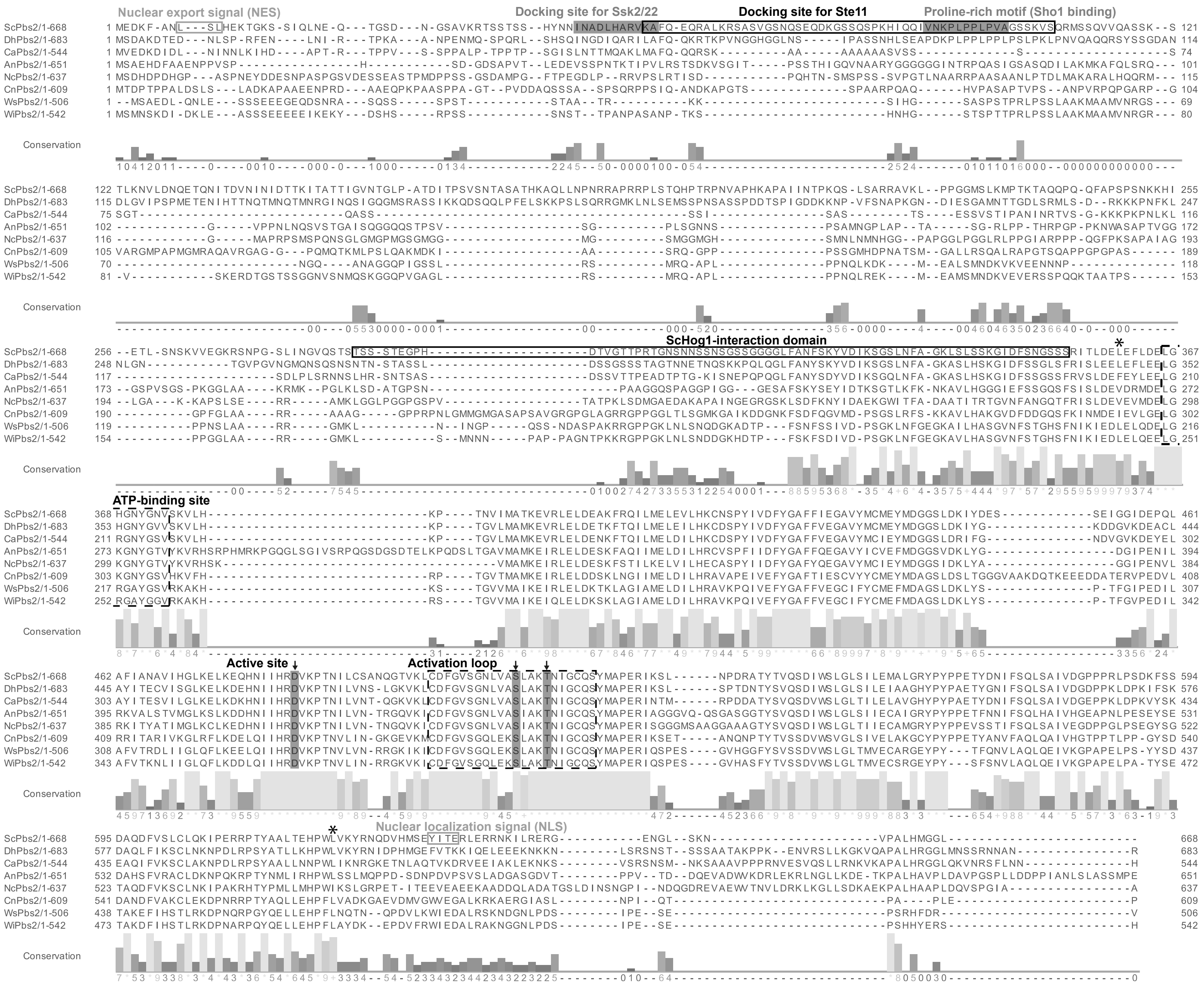

SUPPLEMENTAL FIGURE S1. Protein alignment of selected orthologous Pbs2 kinases. Prefixes indicate the source organism of Pbs2, as in Figure 2. Important domains, motifs and sites are highlighted. Framed boxes, ScPbs2 nuclear export signal, docking site for ScSte11, ScHog1-interaction domain, and ScPbs2 nuclear localization signal; gray boxes, ScSsk2/ScSsk22 docking site and proline-rich motifs for ScSho1 binding; asterisks, start and finish of the kinase domain; dashed boxes, ATP-binding site, and activation loop containing the SLA(I)KT motif; black arrows and gray boxes, S and T phosphorylation site residues in the activation loop, and active site. The columns demonstrate conservation of amino acids (higher and lighter, greater conservation). See Supplemental Table S3 for the GenBank accession numbers.
