# Supplementary material for: Reconstruction of the High-Osmolarity Glycerol (HOG) Signaling Pathway from the Halophilic Fungus Wallemia ichthyophaga in Saccharomyces cerevisiae
Source: Front Microbiol. 2016 Jun 13;7:901. doi: 10.3389/fmicb.2016.00901 (PMC4904012; doi:10.3389/fmicb.2016.00901)
Supplement: Supplementary file 2 [file Image2.PDF]

Supplementary Material

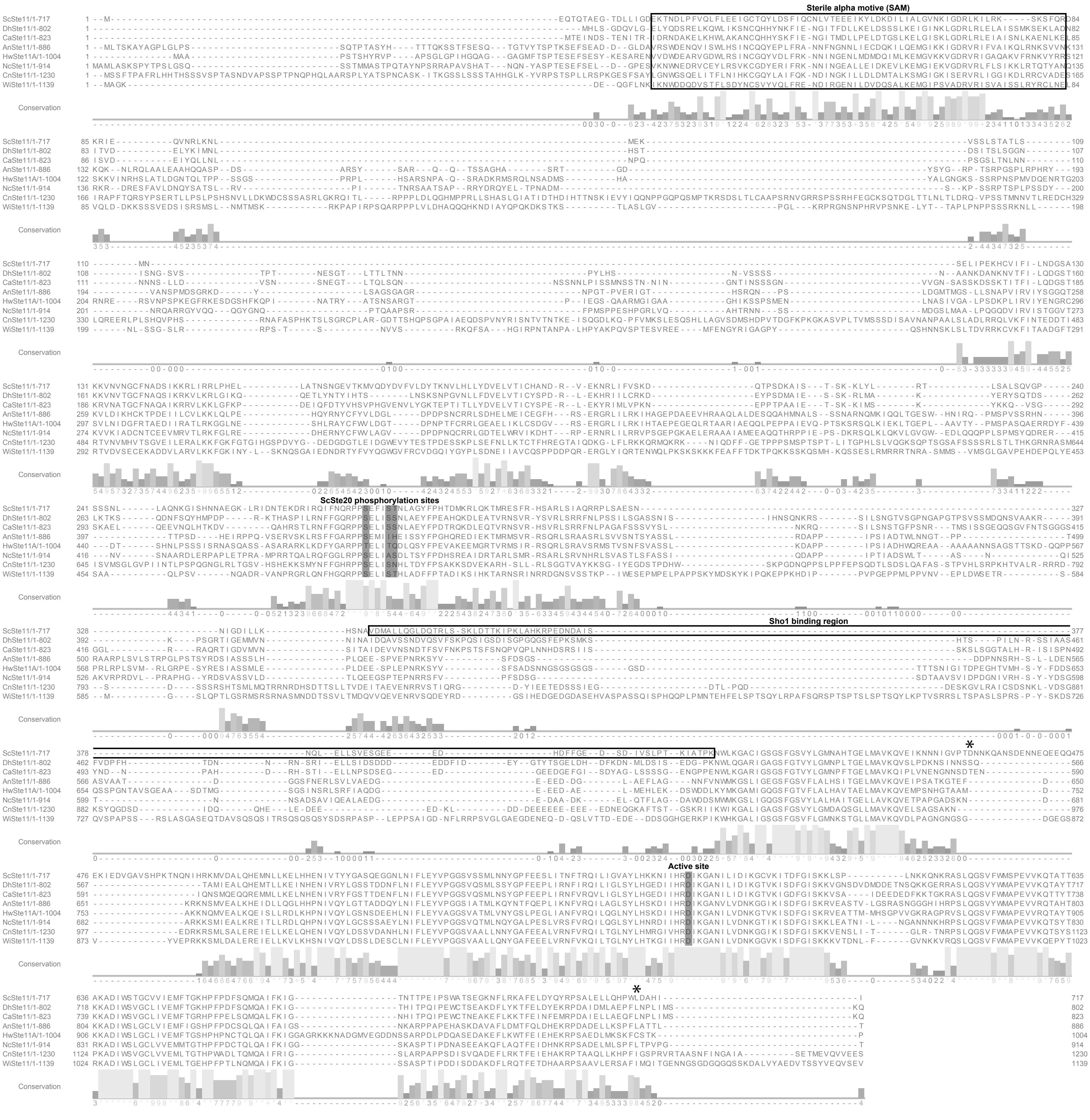

SUPPLEMENTAL FIGURE S2. Protein alignment of selected orthologous Ste11 kinases. Prefixes indicate the source organism of Pbs2, as in Figure 2. Important domains, motifs and sites are highlighted. Framed boxes, Sterile alpha motif and Sho1 binding region; gray boxes, ScSte20 phosphorylation sites, and active site; asterisks, start and finish of the kinase domain. The columns demonstrate conservation of amino acids (higher and lighter, greater conservation). GenBank accession numbers: AnSte11, CAD44493; CaSte11, KGU15182; CnSte11 serotype, AAN75716; DhSte11, CAG85175; NcSte11, EAA33758; ScSte11, AAB67571; WiSte11, EOQ99709. HwSte11: sequence not published.
