# Supplementary material for: Reconstruction of the High-Osmolarity Glycerol (HOG) Signaling Pathway from the Halophilic Fungus Wallemia ichthyophaga in Saccharomyces cerevisiae
Source: Front Microbiol. 2016 Jun 13;7:901. doi: 10.3389/fmicb.2016.00901 (PMC4904012; doi:10.3389/fmicb.2016.00901)
Supplement: Supplementary file 3 [file Image3.PDF]

Supplementary Material

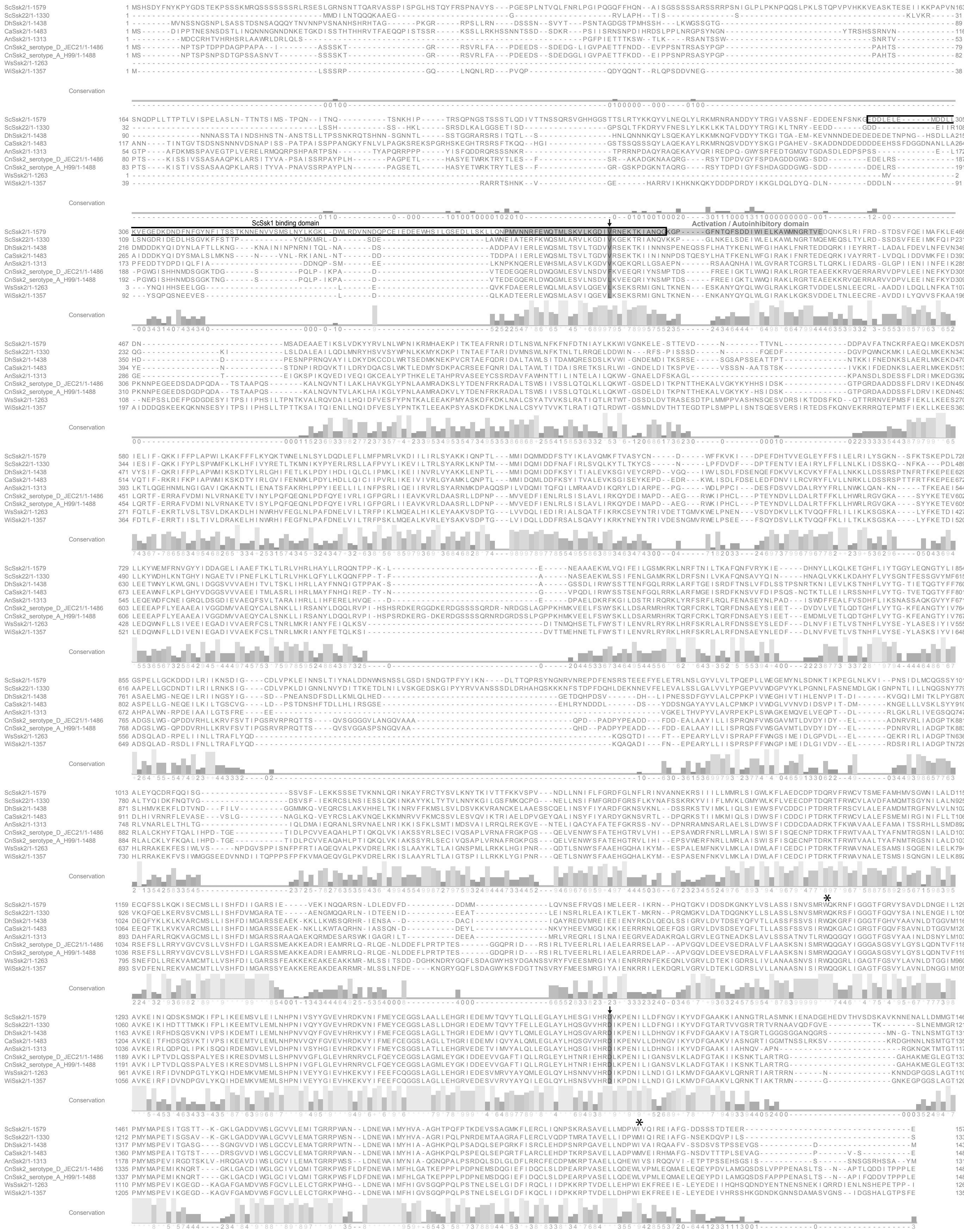

SUPPLEMENTAL FIGURE S3. Protein alignment of selected orthologous Ssk2 kinases. Prefixes indicate the source organism of Pbs2, as in Figure 2. Important domains, motifs and sites are highlighted. Framed box, ScSsk1 binding domain; gray box and arrow, homologous positions in CnSsk2 MAPKKKs with the change of Phe240 to Leu, and active site; light gray box, activation/ autoinhibitory domain; asterisks, start and finish of the kinase domain. The columns demonstrate conservation of amino acids (higher and lighter, greater conservation). GenBank accession numbers: AnSsk2, CBF87987; CaSsk2, KHC76590; CnSsk2 serotype A H99, AFR94327; CnSsk2 serotype D JEC21, AAW46575; DhSsk2, CAG86135; ScSsk2, CAA96311; ScSsk22, CAY78276; WiSsk2, EOR00620; WsSsk2, EIM20776.
