# Supplementary material for: Reconstruction of the High-Osmolarity Glycerol (HOG) Signaling Pathway from the Halophilic Fungus Wallemia ichthyophaga in Saccharomyces cerevisiae
Source: Front Microbiol. 2016 Jun 13;7:901. doi: 10.3389/fmicb.2016.00901 (PMC4904012; doi:10.3389/fmicb.2016.00901)
Supplement: Supplementary file 4 [file Table1.DOCX]

**Supplemental Table S1.** *S. cerevisiae* strains used in this study.

| **Strain** | **Name used here** | **Source** | **Genotype** |
| --- | --- | --- | --- |
| W303 | WT | Euroscarf | Mat a; his3; leu2; trp1; ura3 |
| Y00000 (S288c) | WT | Euroscarf | BY4741; Mat a; his3D1; leu2D0; met15D0; ura3D0 |
| S1278b | WT | Kentaro Furukawa, Göteborg | MATalpha leu2::hisG trp1::hisG his3::hisG ura352 |
| S1278b*FUS1lacZ* | WT | This study ^a^ | MATalpha leu2::hisG trp1::hisG his3::hisG ura352:NatNT2+FUS1lacZ |
| Y07101 | *pbs2Δ* | Euroscarf | BY4741; Mat a; his3D1; leu2D0; met15D0; ura3D0; YJL128c::kanMX4 |
| AZ116 | *sho1Δssk2/22Δ* | Lim Lab, San Francisco | MATa ura3 leu2 his3 trp1 sho1::hisG, ssk2::HIS3-Cg, ssk22::HIS3Cg |
| AZ117 | *sho1Δssk2/22Δpbs2Δ* | Lim Lab, San Francisco | MATa ura3 leu2 his3 trp1 sho1::hisG, ssk2::HIS3-Cg, ssk22::HIS3Cg, pbs2::KAN |
| TM280 | *ssk2/22Δpbs2Δ* | Francesc Posas, Barcelona | MATa ura3 leu2 trp1 ssk2::LEU2, ssk22::LEU2, pbs2::URA3 |
| TM280*FUS1lacZ* | *ssk2/22Δpbs2Δ*  *FUS1-lacZ* | This study ^a^ | MATa ura3 leu2 trp1 ssk2::LEU2, ssk22::LEU2, pbs2::URA3, his3::hphNT1+FUS1lacZ |
| KT005 | *ste11Δpbs2Δ* | Haruo Saito, Tokyo | MATa ura3 leu2 trp1 his3 ste11::HIS3, pbs2::LEU2 |
| 2056 | *ste11Δssk2/22Δ* | Enikö Zorgo, Göteborg | MATa leu23/112 ura31 trp11 his311/15 ade21 can1100 GAL SUC2 ssk2D::KanMX ssk22D::KanMX |
| 1891 | *hog1Δpbs2Δ* | Kentaro Furukawa, Göteborg | MATalpha leu2::hisG trp1::hisG his3::hisG ura352 hog1D::TRP1 pbs2D::KanMX |
| 1891*FUS1 lacZ* | *hog1Δpbs2Δ*  *FUS1-lacZ* | This study ^a^ | MATalpha leu2::hisG trp1::hisG his3::hisG ura352:NatNT2+FUS1lacZ hog1D::TRP1 pbs2D::KanMX |

^a^ see section “*The Cross-Talk β-Galactosidase Assay*” for details of *FUS1-lacZ* strain construction

WT, wild-type
