# Supplementary material for: Reconstruction of the High-Osmolarity Glycerol (HOG) Signaling Pathway from the Halophilic Fungus Wallemia ichthyophaga in Saccharomyces cerevisiae
Source: Front Microbiol. 2016 Jun 13;7:901. doi: 10.3389/fmicb.2016.00901 (PMC4904012; doi:10.3389/fmicb.2016.00901)
Supplement: Supplementary file 5 [file Table2.DOCX]

**Supplemental Table S2.** Primers used in this study.

| **Name  ^a^** | **Sequence (5' – 3') ^b^** |
| --- | --- |
| pWiPBS2.f | ATATGTGATTTCGGTGTATCGGGACAACT |
| pWiPBS2.r | CGAGAAGTCACTAGCCAAGACCAACAT |
| npWiPBS2.f | CCAATTCCATAGCAATACCTGCGAGTT |
| npWiPBS2.r | TGTATCTCCTTAATTGCCATCACCAC |
| WiPBS2.f | ATGAGCATGAACAGTAAAGATATAGATA |
| WiPBS2.r | CTAATGCGACCTCTCATAATGAT |
| (EcoRI)WiPBS2.f | CCCGAATTCATGAGCATGAACAGTAAAGATATAGATA |
| (SacI)WiPBS2.r.MYC | TATGAGCTCCTACAAATCTTCTTCAGAAATCAATTTTTGTTCATGCGACCTCTCATAATGAT |
| WiPbs2(ScPRO).f | CATTGCCGCCTCTTCCCGTAGCAGGAAGCTTAGCAGCGAAAATGGCA |
| WiPbs2(ScPRO).r | GGAAGAGGCGGCAATGGCTTATTAACCGTCGTTGGCGATGTGCT |
|  |  |
| degWiSTE11.f | GCGAGCTGATGGCCGTIAARCARRT |
| degWiSTE11.r | CGGGCACGTACTCCARRAAIAYRTT |
| pWiSTE11.f | AGGTGCTCAAGCATAGCAATATCGTGC |
| pWiSTE11.r | GACATACACGCTGCCCTCACCATC |
| npWiSTE11.f | TCGTCCCTCGATGAGAGCTGTCTGA |
| npWiSTE11.r | ATCGCCACTTCCATTTCCATTTCC |
| WiSTE11.f | ATGGCCGGGAAGGATGA |
| WiSTE11.r | TTACACCTCGGACACTTGTTCCA |
| (BamHI)WiSTE11.f | TATGGATCCTATGGCCGGGAAGGATGA |
| (HindIII)WiSTE11.f | TCTAAGCTTTTACACCTCGGACACTTGTTCCA |
|  |  |
| WiSHO1.f | ATGAAGTTATTCAACGGTGGTAA |
| WiSHO1.r | CTAAACCATATTCAGGTAATTGGAA |
| (HindIII)WiSHO1.f | AAGAAGCTTATGAAGTTATTCAACGGTGGTAA |
| (SacI)WiSHO1.r | ATTGAGCTCTTACTAAACCATATTCAGGTA |
| (KpnI)SHO.f | GTCGATGGTACCTTCTCTTGTTTATCTGGGGGCTA |
| WiSHO1xSHO.r | GTTGAATAACTTCATATTTTGAAAACGTGA |
| SHOxWiSHO1.f | TCACGTTTTCAAAATATGAAGTTATTCAACGGT |
| GFPxWiSHO1.r | CTTTACTCTCGAGAAGCTTGGATCCAACCATATTCAGGTAATT |
| WiSHO1xGFP.f | GGATCCAAGCTTCTCGAGAGTAAAGGAGAAGAACTT |
| (SacI)GFP.r | ACAGAGCTCCTAATTGTATAGTTCATCCATGCCAT |
| (BamHI)WiSH3.f | TTAGGATCCCATATCCCAGATCACGA |
| (EcoRI)WiSH3.r | GCAGAATTCAACCATATTCAGGTAATT |

^a^ In the primer names, f and r denote the primers which anneal to the non-coding and the coding DNA strands, respectively; p and np denote the primers that were used for primary touchdown PCR and the following secondary nested PCR, respectively.

^b^ Restriction sites shown by the underlined parts of the primer sequences.
