# Supplementary material for: Reconstruction of the High-Osmolarity Glycerol (HOG) Signaling Pathway from the Halophilic Fungus Wallemia ichthyophaga in Saccharomyces cerevisiae
Source: Front Microbiol. 2016 Jun 13;7:901. doi: 10.3389/fmicb.2016.00901 (PMC4904012; doi:10.3389/fmicb.2016.00901)
Supplement: Supplementary file 6 [file Table3.DOCX]

**Supplemental Table S3**. HOG-pathway proteins used as *in-silico* probes for identification of orthologous pathway components in the genome of *W. ichthyophaga*, and the Pbs2 and Sho1 orthologs used in the alignments.

| **Ortholog** | **GenBank accession number** | **Source organism** |
| --- | --- | --- |
| Pbs2 | EGC44143 | *Ajellomyces capsulatus* |
|  | EDP56828 | *Aspergillus fumigatus* |
|  | CBF88502 | *Aspergillus nidulans* |
|  | CCD50168 | *Botryotinia fuckeliana* |
|  | EEQ44642 | *Candida albicans* |
|  | BAI48021 | *Cochliobolus heterostrophus* |
|  | AAW41278 | *Cryptococcus neoformans* |
|  | BAC56235 | *Debariomyces hansenii* |
|  | EHY54368 | *Exophiala dermatitidis* |
|  | EDP44139 | *Malassezia globosa* |
|  | EAW22255 | *Neosartorya fischeri* |
|  | BAC56235 | *Neurospora crassa* |
|  | EEA25512 | *Penicillium marneffei* |
|  | EIE79432 | *Rhizopus delemar* |
|  | CAA89423 | *Saccharomyces cerevisiae* |
|  | EFI95111 | *Schizophyllum commune* |
|  | CAB52609 | *Schizosaccharomyces pombe* |
|  | CBQ72530 | *Sporisorium reilianum* |
|  | EAK82802 | *Ustilago maydis* |
|  | EOR04233 | *Wallemia.ichthyophaga* |
|  | EIM23132 | *Wallemia sebi* |
| Sho1 | EAA61884 | *Aspergillus nidulans* |
|  | CAC81238 | *Candida albicans* |
|  | EAL17634 | *Cryptococcus neoformans* |
|  | CAG85892 | *Debariomyces hansenii* |
|  | ADF81057 | *Hortaea werneckii* |
|  | KHE80647 | *Neurospora crassa* |
|  | DAA07778 | *Saccharomyces cerevisiae* |
|  | KUR62632 | *Wallemia ichthyophaga* |
|  | EIM23725 | *Wallemia sebi* |
| Cdc24 | DAA06945 | *Saccharomyces cerevisiae* |
| Cdc42 | DAA09547 | *Saccharomyces cerevisiae* |
| Cla4 | CAA96216 | *Saccharomyces cerevisiae* |
| Gpd1 | DAA11828 | *Saccharomyces cerevisiae* |
| Gpd2 | DAA10724 | *Saccharomyces cerevisiae* |
| Gpp1 | DAA08494 | *Saccharomyces cerevisiae* |
| Gpp2 | DAA07721 | *Saccharomyces cerevisiae* |
| Hkr1 | AAB30051 | *Saccharomyces cerevisiae* |
| Msb2 | CAA96997 | *Saccharomyces cerevisiae* |
| Msn1 | CAA99135 | *Saccharomyces cerevisiae* |
| Nik1 | BAF47077 | *Cryptococcus neoformans* |
| Opy2 | EEU04318 | *Saccharomyces cerevisiae* |
| Ptc1 | CAA98562 | *Saccharomyces cerevisiae* |
| Ptc2 | DAA07750 | *Saccharomyces cerevisiae* |
| Ptc3 | CAA84876 | *Saccharomyces cerevisiae* |
| Ptp1 | DAA11636 | *Saccharomyces cerevisiae* |
| Ptp2 | CAA99423 | *Saccharomyces cerevisiae* |
| Ptp3 | DAA07735 | *Saccharomyces cerevisiae* |
| Skn7 | DAA06899 | *Saccharomyces cerevisiae* |
| Sln1 | CAA86131 | *Saccharomyces cerevisiae* |
| Ssk1 | CAA97528 | *Saccharomyces cerevisiae* |
| Ssk2 | CAA96311 | *Saccharomyces cerevisiae* |
| Ssk22 | DAA07544 | *Saccharomyces cerevisiae* |
| Ste11 | DAA09666 | *Saccharomyces cerevisiae* |
| Ste20 | AAA35039 | *Saccharomyces cerevisiae* |
| Ste50 | AAA13629 | *Saccharomyces cerevisiae* |
| Stl1 | DAA12366 | *Saccharomyces cerevisiae* |
| Ypd1 | CAA98815 | *Saccharomyces cerevisiae* |
