# Supplementary material for: Reconstruction of the High-Osmolarity Glycerol (HOG) Signaling Pathway from the Halophilic Fungus Wallemia ichthyophaga in Saccharomyces cerevisiae
Source: Front Microbiol. 2016 Jun 13;7:901. doi: 10.3389/fmicb.2016.00901 (PMC4904012; doi:10.3389/fmicb.2016.00901)
Supplement: Supplementary file 7 [file Table4.DOCX]

**Supplemental Table S4**. Plasmids and transformants used in this study.

| **Gene** | **Yeast strain** | **Plasmid and insert** | **Name of transformant** | **Selection medium** |
| --- | --- | --- | --- | --- |
| *WiSHO1* | W303 wild-type | pAZ301 | WT | YNB-URA |
|  | *sho1Δssk2/22Δ* | pAZ301 | EV | YNB-URA |
|  |  | pAZ301+*ScSHO1* | *ScSHO1* | YNB-URA |
|  |  | pAZ301+*WiSHO1* | *WiSHO1* | YNB-URA |
|  |  | pAZ301+*ScSHO1WiSH3* | *ScSHO1WiSH3* | YNB-URA |
| *WiSTE11* | Y00000 wild-type | pYX142 | WT | YNB-LEU |
|  | *ste11Δssk2/22Δ* | pYX142 | EV | YNB-LEU |
|  |  | pYX142+*ScSTE11* | *ScSTE11* | YNB-LEU |
|  |  | pYX142+*WiSTE11* | *WiSTE11* | YNB-LEU |
| *WiPBS2* | Y00000 wild-type | pYX142 | WT | YNB-LEU |
|  | *pbs2Δ* | pYX142 | EV | YNB-LEU |
|  |  | pYX142+*ScPBS2* | *ScPBS2* | YNB-LEU |
|  |  | pYX142+*WiPBS2* | *WiPBS2* | YNB-LEU |
|  | W303 wild-type | YCplac22 | WT | YNB-TRP |
|  | *ssk2/22Δpbs2Δ* | YCplac22 | EV | YNB-TRP |
|  |  | YCplac22+*ScPBS2* | *ScPBS2* | YNB-TRP |
|  |  | YCplac22+*WiPBS2* | *WiPBS2* | YNB-TRP |
|  | W303 wild-type | YCplac22+*FUS1-lacZ* | WT | YNB-TRP |
|  | *ssk2/22Δpbs2ΔFUS1-lacZ* | YCplac22 | EV | YNB-TRP |
|  |  | YCplac22+*ScPBS2* | *ScPBS2* | YNB-TRP |
|  |  | YCplac22+*WiPBS2* | *WiPBS2* | YNB-TRP |
|  | W303 wild-type | YCplac22 | WT | YNB-TRP |
|  | *ste11Δpbs2Δ* | YCplac22 | EV | YNB-TRP |
|  |  | YCplac22+*ScPBS2* | *ScPBS2* | YNB-TRP |
|  |  | YCplac22+*WiPBS2* | *WiPBS2* | YNB-TRP |
| *WiPBS2* | *sho1Δssk2/22Δpbs2Δ* | pYX142 and pAZ301 | EV | YNB-LEU-URA |
| and |  | pYX142+*ScPBS2* and pAZ301 | *ScPBS2* | YNB-LEU-URA |
| *WiSHO1* |  | pYX142+*ScPBS2* and pAZ301+*ScSHO1* | *ScPBS2* + *ScSHO1* | YNB-LEU-URA |
|  |  | pYX142+*WiPBS2* and pAZ301+*ScSHO1WiSH3* | *WiPBS2* + *ScSHO1WiSH3* | YNB-LEU-URA |
|  |  | pYX142+*WiPBS2* and pAZ301 | *WiPBS2* | YNB-LEU-URA |
| *WiPBS2* | S1278b wild-type | YCplac33 and pYX142 | WT | YNB-LEU-URA |
| and | *hog1Δpbs2Δ* | YCplac33 and pYX142 | EV | YNB-LEU-URA |
| *WiHOG1* |  | YCplac33+*ScPBS2* and pYX142+*ScHOG1* | *ScPBS2* + *ScHOG1* | YNB-LEU-URA |
|  |  | YCplac33+*ScPBS2* and pYX142+*WiHOG1A* | *ScPBS2* + *WiHOG1A* | YNB-LEU-URA |
|  |  | YCplac33+*ScPBS2* and pYX142+*WiHOG1B* | *ScPBS2* + *WiHOG1B* | YNB-LEU-URA |
|  |  | YCplac33+*WiPBS2* and pYX142+*ScHOG1* | *WiPBS2* + *ScHOG1* | YNB-LEU-URA |
|  |  | YCplac33+*WiPBS2* and pYX142+*WiHOG1A* | *WiPBS2* + *WiHOG1A* | YNB-LEU-URA |
|  |  | YCplac33+*WiPBS2* and pYX142+*WiHOG1B* | *WiPBS2* + *WiHOG1B* | YNB-LEU-URA |
|  | S1278b *FUS1-lacZ* | YCplac33 and pYX142 | WT | YNB-LEU-URA |
|  | *hog1Δpbs2ΔFUS1-lacZ* | YCplac33 and pYX142 | EV | YNB-LEU-URA |
|  |  | YCplac33+*ScPBS2* and pYX142+*ScHOG1* | *ScPBS2* + *ScHOG1* | YNB-LEU-URA |
|  |  | YCplac33+*ScPBS2* and pYX142+*WiHOG1A* | *ScPBS2* + *WiHOG1A* | YNB-LEU-URA |
|  |  | YCplac33+*ScPBS2* and pYX142+*WiHOG1B* | *ScPBS2* + *WiHOG1B* | YNB-LEU-URA |
|  |  | YCplac33+*WiPBS2* and pYX142+*ScHOG1* | *WiPBS2* + *ScHOG1* | YNB-LEU-URA |
|  |  | YCplac33+*WiPBS2* and pYX142+*WiHOG1A* | *WiPBS2* + *WiHOG1A* | YNB-LEU-URA |
|  |  | YCplac33+*WiPBS2* and pYX142+*WiHOG1B* | *WiPBS2* + *WiHOG1B* | YNB-LEU-URA |
