# Supplementary material for: Reconstruction of the High-Osmolarity Glycerol (HOG) Signaling Pathway from the Halophilic Fungus Wallemia ichthyophaga in Saccharomyces cerevisiae
Source: Front Microbiol. 2016 Jun 13;7:901. doi: 10.3389/fmicb.2016.00901 (PMC4904012; doi:10.3389/fmicb.2016.00901)
Supplement: Supplementary file 8 [file Table5.DOCX]

**Supplemental Table S5.** Alphabetically ordered *W. ichthyophaga* HOG-pathway proteins used in this study, their synoynms and GenBank accession numbers.

| **Protein name** | **Synonym** | **GenBank accession number** |
| --- | --- | --- |
| WiCdc24 | WiScd1 | EOR03906 |
| WiCdc42 |  | EOR00654 |
| WiCla4 | WiShk2 | EOQ98992 |
| WiHog1A |  | AGG39582 |
| WiHog1B |  | AGG39583 |
| WiNik1 | Histidine kinase J | EOR01469 |
| WiPbs2 | WiWis1 | EOR04233 |
| WiPtc1 |  | EOQ99025 |
| WiPtc3 |  | EOQ99024 |
| WiPtp1 |  | KUR62633 |
| WiPtp3 | Tyrosine-protein phosphatase 69D | EOR04868 |
| WiSho1 |  | KUR62632 |
| WiSkn7 | WiPrr1 | EOR04976 |
| WiSsk1 | WiMcs4 | EOQ98783 |
| WiSsk2 | WiWis4 | EOR00620 |
| WiSte11 | WiByr2 | EOQ99709 |
| WiSte20 | WiSmu1 | EOR01184 |
| WiSte50 |  | EOR03947 |
| WiYpd1 |  | EOQ99342 |
